# Leukemic Cells Hijack Stromal Bioelectricity to Reprogram the Bone Marrow Niche via CaV1.2‐Dependent Mechanisms

**DOI:** 10.1002/advs.202508940

**Published:** 2025-08-20

**Authors:** Ambra Da Ros, Maddalena Benetton, Giulia Borella, Giorgia Longo, Giulia Borile, Alice Cani, Diego Lopez‐Pigozzi, Mario Bortolozzi, Silvia Bresolin, Claudia Tregnago, Franco Locatelli, Martina Pigazzi

**Affiliations:** ^1^ Department of Women's and Children's Health, Onco‐Hematology lab and clinic University of Padova Padova 35128 Italy; ^2^ Foundation Istituto Ricerca Pediatrica Città della Speranza Padova 35127 Italy; ^3^ Department of Physics and Astronomy “G. Galilei” University of Padova Padova 35121 Italy; ^4^ Veneto Institute of Molecular Medicine (VIMM) Padova 35129 Italy; ^5^ Department of Pediatric Hematology and Oncology, IRCCS, Ospedale Pediatrico Bambino Gesù Catholic University of the Sacred Heart Rome 00165 Italy

**Keywords:** acute myeloid leukemia, bioelectricity, mesenchymal stromal cells, tumor microenvironment, voltage membrane potential

## Abstract

Mesenchymal stromal cells (MSCs) are key components of the tumor microenvironment (TME), influencing leukemia progression through poorly understood mechanisms. Here, the bioelectrical properties of MSCs derived from pediatric acute myeloid leukemia (AML) patients (AML‐MSCs) are investigated, identifying a significant depolarization of their resting voltage membrane potential (V_mem_, −14.7 mV) compared to healthy MSCs (h‐MSCs, −28.5 mV), accompanied by downregulation of Calcium channel, voltage‐dependent, L type, alpha 1C subunit1.2 (CaV1.2) L‐type calcium channel expression. AML‐MSCs display increased spontaneous calcium oscillations, suggesting altered ion homeostasis. Notably, h‐MSCs exposed to AML blasts undergo a similar V_mem_ depolarization (−11.8 mV) and CaV1.2 downregulation, indicating that leukemic cells actively reprogram MSCs. Functionally, V_mem_ depolarization in h‐MSCs promotes a pro‐leukemic phenotype, whereas hyperpolarization of AML‐MSCs restores a normal behavior. CaV1.2 over‐expression by lentiviral vectors in AML‐MSCs shifts the V_mem_ toward hyperpolarization and partially reverses their leukemia‐supportive properties, in part through CaV1.2 transfer via tunneling nanotubes. These findings reveal that AML blasts impose a bioelectrical signature on MSCs, modulating ion channel activity to sustain a leukemic niche. Targeting this electrical reprogramming through CaV1.2 restoration represents a potential strategy to re‐establish homeostasis in the bone marrow microenvironment.

## Graphical abstract

Leukemic blasts induce membrane depolarization in mesenchymal stromal cells (MSCs), contributing to a dysfunctional niche that favors leukemia progression. Here, it is demonstrated that restoring the expression of CaV1.2 calcium channel in AML‐MSCs repolarizes their membrane potential, reprogramming them toward a healthy phenotype. Repolarized MSCs recover the capacity to support normal hematopoiesis, unveiling a novel strategy to re‐establish bone marrow homeostasis.

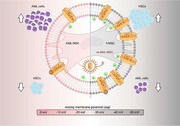

## 1 Introduction

Recent advancements in cancer research indicate that cancer is not only due to malignant cells, but involves a complex network of transformed and non‐transformed cells, including nervous, endothelial, stromal, stem, and immune cells that make up the tumor microenvironment (TME).^[^ ^1^ ^]^ In this context of interconnected tumor cells and TME, how intra‐ or extra‐cellular conditions and ion channels contribute to cancer is largely under‐explored. Ion channels are membrane proteins that can catalyze the selective and regulated diffusion of ions across cellular membranes.^[^ ^2^, ^3^ ^]^ Their activity is mutually integrated into physiological processes, controlling cell signaling pathways and being tightly controlled by them. In addition to well‐recognized ones, like voltage‐gated Na^+^/K^+^/Ca^2+^ channels in heart and neurons, novel ion channels are continuously discovered in both excitable and non‐excitable cells and play important roles in developmental disorders, neurodegenerative diseases, and cancer, constituting the second largest class of drug targets after G protein‐coupled receptors.^[^ ^4^, ^5^ ^]^ Ion channels work in concert to maintain ionic homeostasis and cellular resting voltage membrane potential (V_mem_), which refers to the electric potential difference between cytoplasm and extracellular space. Their “on” or “off” state governance and ability to form electrical networks in response to inputs from local TME is under investigation.^[^ ^6^, ^7^ ^]^ An abnormal depolarization of V_mem_ and ion channels' conductivity has been reported to play a role in cancer initiation, TME remodeling, and therapy response, particularly in solid tumors.^[^ ^8^, ^9^, ^10^ ^]^ Importantly, V_mem_ has an impact on cellular differentiation and proliferation, with depolarization characterizing plastic, undifferentiated, proliferative cells, while increased negative V_mem_ (hyperpolarization) supports cell differentiation and lower proliferation.^[^ ^7^, ^11^ ^]^ As central regulators of cellular electrical properties, ion channels might be implicated in all steps of tumorigenesis. However, in the field of ion channel discovery, there are few published studies, often reporting unconfirmed data.^[^ ^12^ ^]^ Particularly in stem and progenitor cells, including adult human mesenchymal stromal cells (MSCs), a forced depolarization has been shown to maintain cells in a stem‐cell like state,^[^ ^13^, ^14^, ^15^, ^16^ ^]^ and the identification of a V_mem_ threshold to separate “normal” quiescent or resting cells from proliferative or cancerous ones is of great interest. Furthermore, observations that hyperpolarizing treatments inhibit tumor formation suggest that depolarization is a disease phenotype that is worth investigating,^[^ ^17^ ^]^ and that the reversible nature of bioelectric tissue remodeling supports the development of innovative therapies,^[^ ^18^, ^19^, ^20^ ^]^ with ion channels being the cancer molecular targets of ≈15% of US FDA‐approved drugs.^[^ ^21^, ^22^, ^23^ ^]^

Pediatric cancer is still a life‐threatening disease with few new drugs in the market, particularly for acute leukemia, the most frequent tumor in childhood.^[^ ^24^, ^25^ ^]^ We recently attributed for the first time a role to calcium signaling and ion channels in MSCs within the leukemic bone marrow (BM) niche. Using our previously established 3D BM niche model, we discovered that Lercanidipine, Calcium channel, voltage‐dependent, L type, alpha 1C subunit1.2 (CaV1.2) blocker, induces functional modifications in acute myeloid leukemia (AML)‐derived MSCs.^[^ ^26^ ^]^ This highlights a previously unrecognized mechanism by which ion channel activity in MSCs contributes to shaping the TME and supporting leukemia progression.

Here, we documented that non‐excitable MSCs possess a resting V_mem_ that undergoes significant depolarization upon exposure to leukemic cells (called blasts). This shift is driven by altered expression of CaV1.2, a key regulator of calcium influx, which in turn promotes novel pro‐leukemic functions that shape the TME.

## 2 Results

### 2.1 Ca^2+^ Flux Dynamics are Altered in Depolarized AML‐MSCs

Experiments throughout the manuscript were performed by using primary MSCs derived from primary AML at diagnosis or healthy donors (*n* = 54 and *n* = 26 respectively, Table S1, Supporting Information).

**Figure 1:**
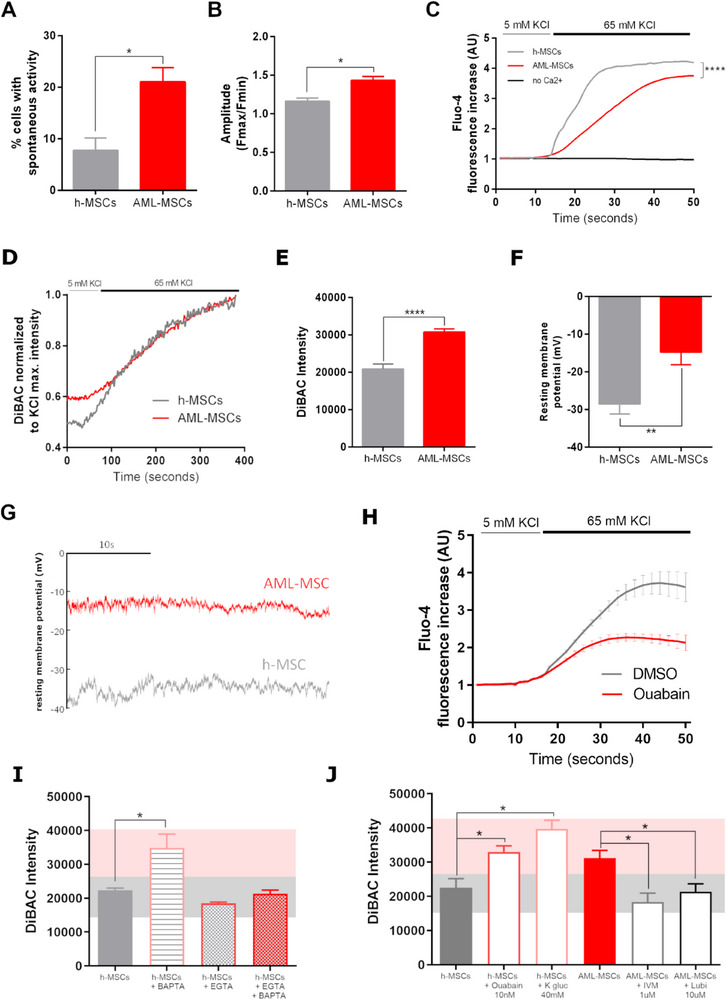
Ca^2+^ flux and resting V_mem_ are altered in AML‐MSCs. A) Percentage of spontaneous Ca^2+^ oscillating h‐MSCs and AML‐MSCs cells loaded with calcium indicator Fluo‐4 AM probe (*n* = 3, *t*‐test). B) Average amplitude of calcium oscillations in h‐MSCs and AML‐MSCs loaded with Fluo‐4 AM probe (*n* = 3, 7, and 50 cells per sample, respectively, *t*‐test). C) Intracellular calcium increases in h‐MSCs and AML‐MSCs, loaded with Fluo‐4 AM probe and stimulated with KCl (65 mm), in the presence/absence of external Ca^2+^ (*n* = 3, *t*‐test at maximum h‐MSCs fluorescence increase. D) DiBAC fluorescence intensity in h‐MSCs and AML‐MSCs loaded with the voltage‐sensitive dye DiBAC and stimulated with KCl (65 mm). Fluorescence intensity data were normalized to the KCl maximum intensity value (*n* = 4, *t*‐test at 200, 300, and 400 s, *p *= not significant). E) Fluorescence intensity calculated from DiBAC fluorescence staining in AML‐MSCs (*n* = 14) and h‐MSCs (*n* = 7) (25 cells/image, 4 images, *t*‐test). F) Histogram of average resting membrane potentials (mV) measured by patch clamp in AML‐MSCs (*n* = 5, 11 cells) and h‐MSCs (*n* = 4, 10 cells, *t*‐test). G) Representative traces of two cells where resting membrane potential was recorded in current clamp configuration. H) Intracellular calcium increases in h‐MSCs treated or not with the depolarizing agent Ouabain 10 nm, and then loaded with Fluo‐4 AM probe and stimulated with KCl (*n* = 4, 100 cells, AU: arbitrary unit, *t*‐test performed at every other second, being significant from 30 to 50 s). I) DiBAC fluorescence intensity in h‐MSCs treated with BAPTA (50 µm) or EGTA (2 mm) alone or in combination (*n* = 3, *t*‐test comparing all treated groups versus h‐MSCs). J) DiBAC fluorescence intensity in h‐MSCs treated with Ouabain (10 nm, *n* = 4) or K^+^ gluconate (40 mm, *n* = 3, *t*‐test comparing treated groups versus h‐MSCs) and in AML‐MSCs treated with Ivermectin (IVM, 1 µm, *n* = 3) or Lubiprostone (Lubi, 10 µm, *n* = 5, *t*‐test comparing treated groups versus AML‐MSCs) for 72 h. All histograms show mean ± SEM; ^*^ *p*  < 0.05, ^**^ *p* < 0.01, ^****^ *p* < 0.0001.

We previously characterized MSCs derived from pediatric AML samples collected at diagnosis (AML‐MSCs), highlighting pro‐oncogenic features (proliferation, differentiation capability, transcriptome, and secretome) and reduced CaV1.2 expression with respect to MSCs derived from age‐matched healthy donors (h‐MSCs). This latter finding resulted in an increased sensitivity of AML‐MSCs to the Ca^2+^‐channel blocker Lercanidipine.^[^ ^26^ ^]^ This observation suggested that Ca^2+^ homeostasis could play a critical role in modifying MSCs behavior within the BM niche. Using Fluo4‐AM in h‐MSCs and AML‐MSCs, we observed that AML‐MSCs increased spontaneous Ca^2+^ oscillations (*p *< 0.05, **Figure** **1**A), which had a higher amplitude than in h‐MSCs (*p *< 0.05, Figure 1B). In addition, in response to high‐KCl depolarization stimulus, AML‐MSCs significantly reduced their extracellular Ca^2+^ uptake with respect to h‐MSCs (*p *< 0.0001, Figure 1C). Since several works suggested that changes in intracellular Ca^2+^ handling are associated with CaV1.2 and V_mem_,^[^ ^9^, ^27^ ^]^ we investigated MSCs V_mem_ by loading AML‐ and h‐MSCs with the voltage‐sensitive dye DiBAC, whose uptake is higher in depolarized cells, in response to KCl depolarization stimulus. Results showed that AML‐MSCs showed a different DiBAC fluorescence intensity, uncovering a different resting V_mem_ (Figure 1D) and, upon depolarizing KCl stimulus, DiBAC fluorescence increased in a time‐dependent manner. Thus, we measured V_mem_ of several h‐MSCs (*n* = 7) and AML‐MSCs (*n* = 14), confirming AML‐MSCs depolarization compared to h‐MSCs (*p *< 0.0001, Figure 1E; Figure S1A, Supporting Information). We documented that h‐MSCs V_mem_ ranged from 15500 to 26000 (DiBAC mean pixel intensity), whereas AML‐MSCs ranged from 26000 to 38500, at day 3 of culture (Table S2, Supporting Information). We validated V_mem_ measurements by patch clamp, confirming AML‐MSCs depolarization for h‐MSCs (V_mem_ average = −14.7 mV ± 3.36 mV and −28.5 mV ± 2.58 mV, respectively, *p *< 0.01, Figure 1F,G; Figure S1B, Supporting Information). These latter findings showed, to the best of our knowledge, for the first time, that non‐excitable MSCs acquire a different V_mem_ at leukemia onset, rendering them AML‐MSCs.

To determine the contribution of Ca^2+^ dynamics in MSCs depolarization, we treated h‐MSCs with Ouabain, a Na^+^/K^+^‐ATPase inhibitor that induces a tonic depolarization, observing that Ca^2+^ uptake was significantly lowered compared to untreated cells (Figure 1H), confirming an association between membrane depolarization and Ca^2+^ levels. In addition, we treated h‐MSCs with the Ca^2+^ selective intracellular and extracellular chelators, BAPTA‐AM and EGTA, respectively, and monitored V_mem_ changes, showing that treatment with BAPTA shifted DiBAC intensity toward positive voltages‐depolarization (*p *< 0.05, Figure 1I), whereas in the presence of EGTA, which blocks Ca^2+^ influx, V_mem_ remained constant. These results demonstrate V_mem_ dependence on Ca^2+^ influx dynamics and support a bi‐directional interplay between Ca^2+^ handling, the central coordinator of excitable cells' functions, and MSCs depolarization.

We modulated V_mem_ by ion channel blockers or activators to promote bioelectric changes without affecting cell viability, and simultaneously tracked the corresponding voltage changes. H‐MSCs were treated with the depolarizing agent Ouabain at 5, 10, 25 nm, or with K^+^ gluconate, a blocker of K^+^ channels, at 10, 40, 80 mm, for 3 days. Both treatments induced depolarization with increasing concentration (Figure S1C,D, Supporting Information). In particular, the depolarizing treatment with 10 nm Ouabain or alternatively 40 mm K^+^ gluconate displayed a time‐dependent effect, reaching a V_mem_ similar to that of AML‐MSCs (leukemic interval‐pink band, *p *< 0.05, Figure 1J), and after 3 days of drug washout V_mem_ maintained depolarized status (Figure S1E, Supporting Information).

On the contrary, we modulated AML‐MSCs V_mem_ by using hyperpolarizing agents, Ivermectin (IVM), an activator of glutamate‐gated chloride channels, at 0.1 and 1 µm, and Lubiprostone (Lubi), an activator of ClC‐2 chloride channels, at 1, 10, 50 µm, for 3 days. Interestingly, we found that DiBAC fluorescence significantly decreased with 10 µm of Lubi or with 1 µm of IVM from 24 h of treatment, and persisted after 72 h and after the washout, indicating AML‐MSCs V_mem_ hyperpolarization by drug exposure (healthy interval‐grey band, *p *< 0.05, Figure S1F–H, Supporting Information).

### 2.2 MSCs V_mem_ Pharmacological Depolarization Induced Functional Reprogramming

**Figure 2:**
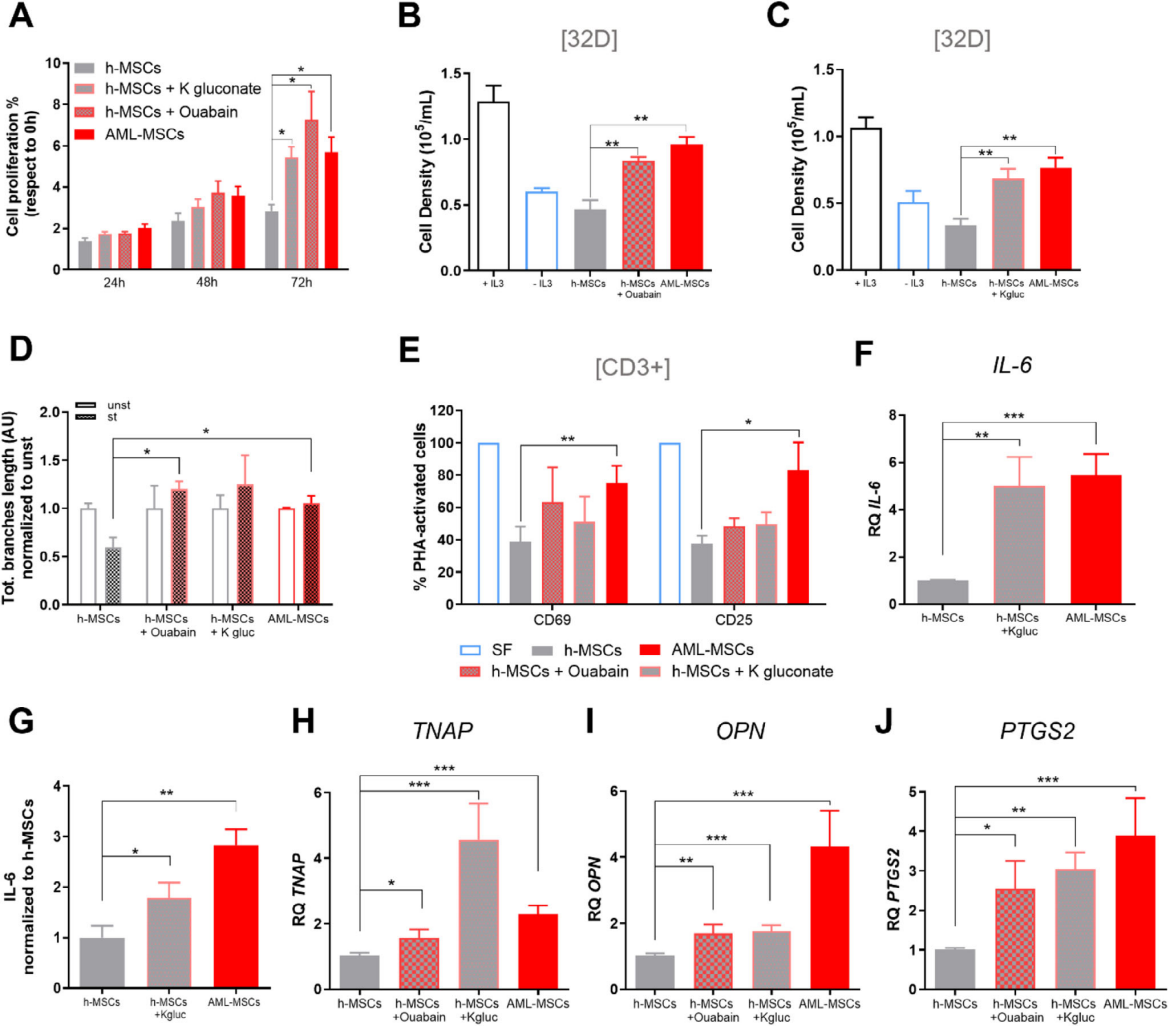
V_mem_ pharmacological depolarization induces an AML‐MSCs‐like phenotype in h‐MSCs. A) Cell proliferation of AML‐MSCs, h‐MSCs, and h‐MSCs treated with 40 mm K^+^ gluconate or 10 nm Ouabain by Presto Blue assay (*n* = 5). Data were normalized to time 0‐hour samples (*t*‐test comparing all groups versus h‐MSCs). B,C) Cell density of murine IL‐3–dependent 32D cell line cultured in the presence (black bar) or absence of IL‐3 (blue bar), compared with 32D cell line cultured on a layer of AML‐MSCs (red bar), h‐MSCs (grey) or h‐MSCs pre‐treated for 72 h with Ouabain (B, *n* = 5) or with K^+^ gluconate (C, *n* = 5). *t*‐test was performed to compare treated groups or AML‐MSCs versus h‐MSCs, with ±IL3 used as experimental control. D) Total branches length of HUVEC tubes by using conditioned medium derived from AML‐MSCs (*n* = 7) and h‐MSCs pre‐treated or not for 72 h with Ouabain or K^+^ gluconate and then stimulated (st) or not (unst) with a pro‐inflammatory cytokine cocktail (hIL‐1β, hIL‐6, and hTNF‐α) for 24 h; tube formation was evaluated after 4 h and normalized to unst condition (AU: arbitrary unit, *n* = 4, *t*‐test comparing treated or AML‐MSCs stimulated groups versus stimulated h‐MSCs). E) Percentage of PHA‐stimulated CD3^+^ *T*‐cells expressing CD69 and CD25 after 72 h of co‐culture with AML‐MSCs and h‐MSCs pre‐treated for 72 h with Ouabain or K^+^ gluconate, relative to SF condition (without MSCs) (*n* = 4, *t*‐test comparing treated or AML‐MSCs groups versus h‐MSCs). F) Relative expression measured by RQ‐PCR of *IL‐6* (interleukin‐6) in h‐MSCs pre‐treated or not for 72 h with K^+^ gluconate (*n* = 4) and in AML‐MSCs (*n* = 3, *t*‐test comparing treated or AML‐MSCs groups versus h‐MSCs). G) IL‐6 protein secretion levels (pg/mL), measured by ELISA, in AML‐MSCs (*n* = 24) or in h‐MSCs pre‐treated or not for 72 h with K^+^ gluconate (*n* = 4 or *n* = 6, respectively), relative to h‐MSCs untreated condition (t‐test comparing treated or AML‐MSCs groups versus h‐MSCs). H–J) Relative RQ‐PCR expression of osteoprogenitor‐associated genes *TNAP* (Tissue‐nonspecific alkaline phosphatase, H, *n* = 7 h‐MSCs and *n* = 3 AML‐MSCs) and *OPN* (osteopontin, **I**, *n* = 6 h‐MSCs and *n* = 3 AML‐MSCs), and pro‐inflammatory gene *PTGS2* (prostaglandin‐endoperoxide synthase 2, J), *n* = 7 h‐MSCs and *n* = 5 AML‐MSCs) in h‐MSCs pre‐treated or not for 72 h with Ouabain or K^+^ gluconate and in AML‐MSCs. *T*‐test was used to compare the treated or AML‐MSCs groups versus h‐MSCs. All histograms show mean ± SEM; ^*^ *p* < 0.05, ^**^ *p* < 0.01, ^***^ *p* < 0.001.

Considering these findings, we hypothesized that depolarization of MSCs could be the “first hit” to transform MSCs at leukemia onset, inducing their reprogramming as we previously documented.^[^ ^26^ ^]^ To address this hypothesis, we investigated MSCs functions in vitro after exposure to depolarizing agents. In detail, we observed that depolarizing treatments induced a higher h‐MSCs proliferation (Ouabain, 2.5‐fold; K^+^ gluconate, 1.9‐fold, *p *< 0.05, **Figure** **2**A). Furthermore, depolarized MSCs supported the cytokine‐dependent 32D cell growth without IL‐3 (Figure 2B,C) at the same extent of AML‐MSCs, suggesting that depolarization induced novel MSCs capabilities. Moreover, we documented 32D cell differentiation after co‐culture with AML‐MSCs or h‐MSCs treated with depolarizing agents, shown by a reduced nuclear size, enlarged cytoplasm, and a loss of dark‐violet staining, when compared to h‐MSCs or with standard IL‐3 (Figure S2, Supporting Information). Then, considering that we previously documented an impaired AML‐MSCs anti‐inflammatory potential compared to h‐MSCs,^[^ ^26^ ^]^ we confirmed a reduction of h‐MSCs immunomodulation activity after the exposure to depolarizing agents, as observed in the positive control AML‐MSCs (*p *< 0.05, Figure 2D). Accordingly, PHA‐activated CD3^+^ *T*‐cells cultured on a layer of h‐MSCs reduced their CD69 and CD25 expression, whereas the depolarized h‐MSCs and the AML‐MSCs did not induce down‐regulation of these *T*‐cell activation markers, supporting the hypothesis that depolarization reduced MSCs immunomodulatory properties (Figure 2E). Additionally, we documented *IL‐6* gene over‐expression in the depolarized h‐MSCs (*p *< 0.01, Figure 2F), together with a significantly higher release of IL‐6 in h‐MSCs treated with K^+^ gluconate (1.7‐fold, *p *< 0.05, Figure 2G). In addition, we monitored h‐MSCs expression of key osteo‐progenitor genes after depolarization treatment, finding a significant increase in *TNAP* and *OPN* expression levels as observed in AML‐MSCs (Figure 2H,I). In depolarized h‐MSCs we observed a significant up‐regulation of the inflammation‐related *PTGS2* gene (Figure 2J), previously identified as the top differentially over‐expressed gene between h‐MSCs and AML‐MSCs^[^ ^26^ ^]^. Altogether, V_mem_ depolarization drives a reprogrammed stroma toward the enhancement of an inflamed leukemic niche.

### 2.3 AML Blasts Depolarizes MSCs V_mem_ by Cell–Cell Contact

**Figure 3:**
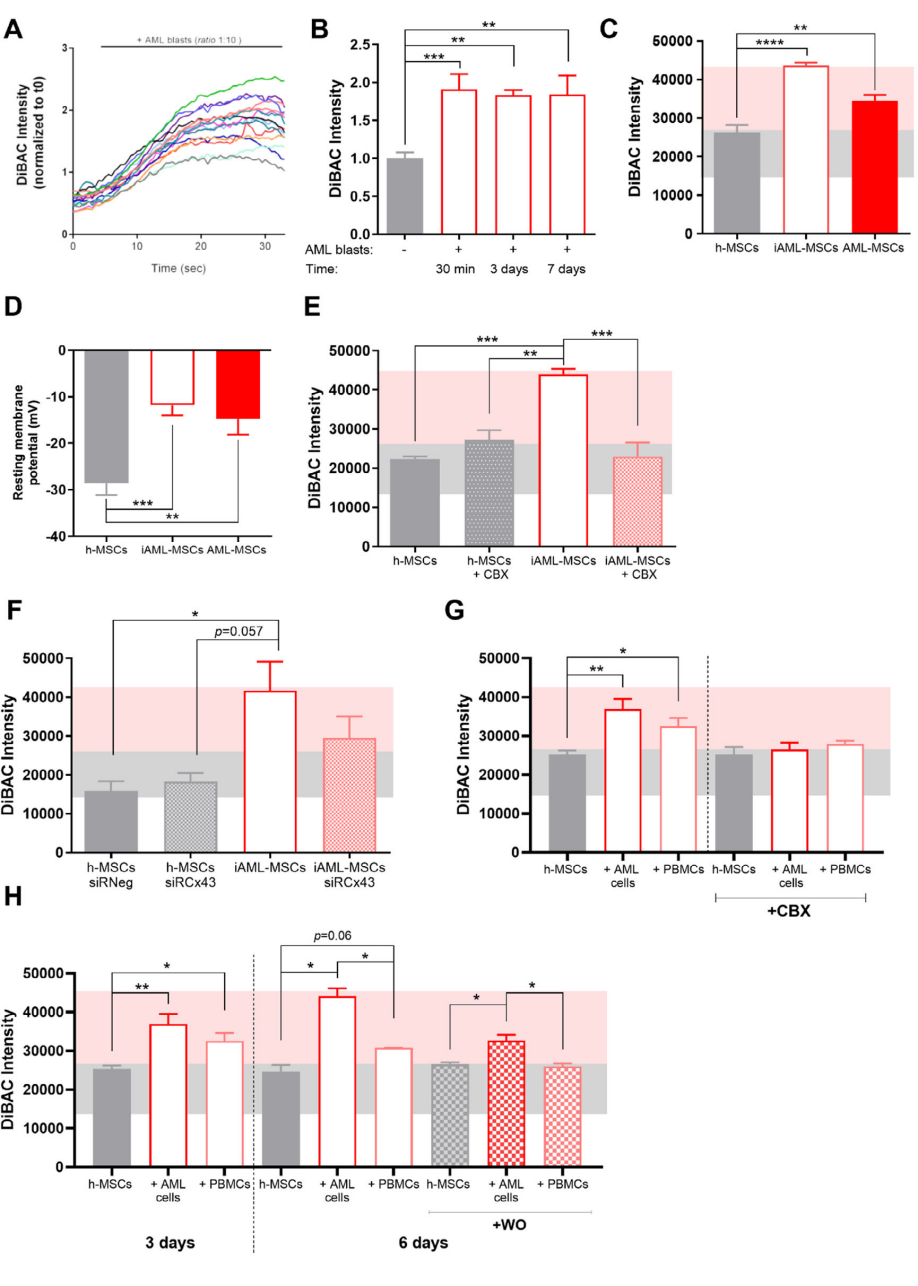
AML blasts depolarize MSCs V_mem_ by cell–cell contact. A) Time course of DiBAC fluorescence intensity in h‐MSCs during co‐culture with AML blasts (ratio 1:10) calculated for the first 30 min of co‐culture and normalized with respect to t0 fluorescence intensity value (15 cells, *n* = 1). B) V_mem_ variations measured by DiBAC fluorescence intensity in h‐MSCs co‐cultured with AML blasts for 30 min, 3 days or 7 days (*n* = 3, *t*‐test comparing all groups versus h‐MSCs). DiBAC fluorescence intensity of h‐MSCs cultured alone was used as control. C) DiBAC fluorescence intensity in h‐MSCs (*n* = 9), h‐MSCs cultured with AML blasts for 4 days (iAML‐MSCs, *n* = 15), and AML‐MSCs (*n* = 11). Pink and grey bands represent the interval values of AML‐ and h‐MSCs DiBAC intensity, respectively, after 4 days of culture. *T*‐test was used to compare i‐AML‐MSCs or AML‐MSCs versus h‐MSCs. D) Histogram of average resting membrane potentials (mV) measured by patch clamp in h‐MSCs (*n* = 4, 10 cells), iAML‐MSCs (*n* = 4, 10 cells), and AML‐MSCs (*n* = 5, 11 cells). *T*‐test was used to compare i‐AML‐MSCs or AML‐MSCs versus h‐MSCs. E,F) DiBAC fluorescence intensity in iAML‐MSCs treated with CBX (100 µm, *n* = 3) or silenced for Cx‐43 (*n* = 3) during co‐culture with AML blasts. h‐MSCs siRNeg and siRCx‐43 DiBAC fluorescence intensity were used as controls. *T*‐test was performed to compare h‐MSCs or i‐AML‐MSCs treated with CBX/SirCx43 versus i‐AML‐MSCs. G) Fluorescence intensity measured by DiBAC staining after 3 days of h‐MSCs co‐culture with AML primary cells or normal PBMCs (*n* = 7), with or without the addition of CBX (*n* = 3). *T*‐test was performed to compare all groups with their respective h‐MSCs group. **H)** DiBAC intensity of h‐MSCs co‐culture with AML primary cells or normal PBMCs for 3 days (*n* = 7), 6 days (*n* = 3), or 3 days of co‐culture followed by 3 days of washout (WO, *n* = 3). *T*‐test used to compare all groups with their respective h‐MSCs group. All histograms show mean ± SEM; ^*^ *p* < 0.05, ^**^ *p* < 0.01, ^***^ *p* < 0.001, ^****^ *p* < 0.0001.

Considering that AML‐MSCs are depolarized compared to h‐MSCs, it is reasonable to speculate that AML blasts play a role in modifying V_mem_ of MSCs residing in the BM niche. We examined the V_mem_ of h‐MSCs after co‐culturing with AML blasts (namely induced AML‐MSCs, iAML‐MSCs) at different timepoints, observing that iAML‐MSCs quickly increased their V_mem_, which remained constant over time reaching the depolarized V_mem_ levels of AML‐MSCs (−11.8 mV, *p *< 0.001, **Figure** **3**A–D; Figure S3A, Supporting Information). To confirm that the contact between blasts and MSCs is responsible for V_mem_ changes, we demonstrated that the addition of a transwell insert in the co‐culture induced a lower V_mem_ change compared to direct cell–cell contact (Figure S3B, Supporting Information). We then focused on Connexin(Cx)‐43‐based gap junctions, fundamental in excitable tissues by facilitating transmission of electric signals between adjacent cells.^[^ ^28^ ^]^ We previously demonstrated that Cx‐43 was responsible for AML‐MSCs transcriptional reprogramming;^[^ ^26^ ^]^ Here we blocked Cx‐43‐mediated cell–cell contact between h‐MSCs and blasts by treating with a gap junction blocker, Carbenoxolone (CBX), or silencing both cell types with Cx‐43 small interfering (si)RNA, and monitored V_mem_. Both strategies inhibiting cell–cell contact blocked MSCs‐bioelectric shift toward a depolarized status (Figure 3E,F). To check if AML blasts exposure was responsible for MSCs V_mem_ changes, excluding effects derived from physiological communication by heterotypic cell–cell contact, we co‐cultured h‐MSCs with either blasts or normal mononuclear cells (PBMCs) for 3 days, indicating that cell–cell contact alters the V_mem_ regardless of cell type, while the addition of CBX prevented this V_mem_ shift (Figure 3G). However, only AML blasts were able to depolarize h‐MSCs up to 6 days of co‐culture and after blast removal (Figure 3H), providing evidence that AML blasts directly induced the MSCs bioelectric shift toward depolarization.

### 2.4 MSCs V_mem_ Pharmacological Hyperpolarization Restores Healthy Features

**Figure 4:**
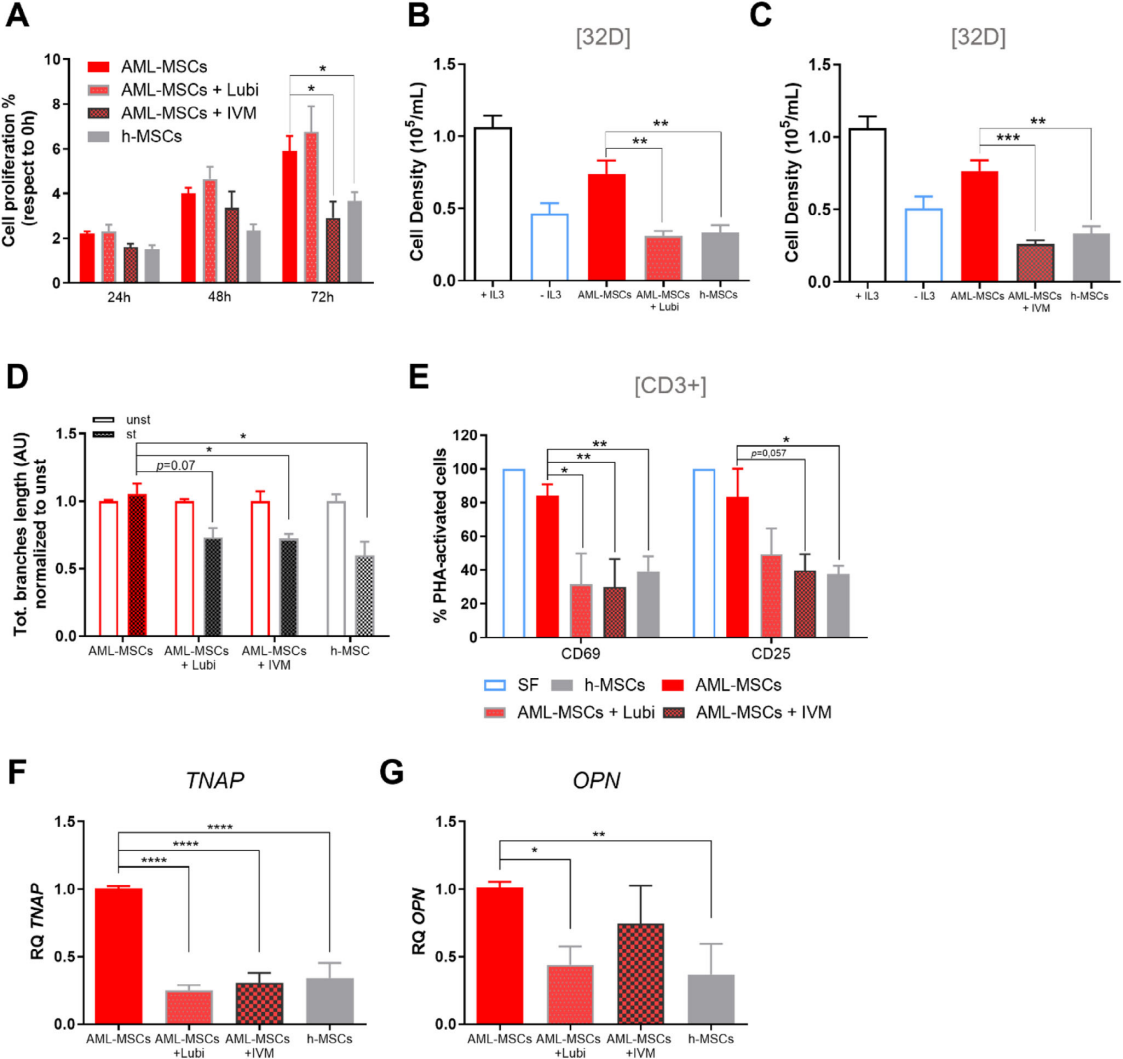
AML‐MSCs V_mem_ pharmacological hyperpolarization restores a healthy phenotype. A) Cell proliferation measured by Presto Blue assay in h‐MSCs and AML‐MSCs treated or not with 10 µm Lubi or 1 µm IVM for 72 h (*n* = 4, *t*‐test comparing all groups versus AML‐MSCs). B,C) Cell density of murine IL‐3–dependent 32D cell line cultured in the presence or absence of IL‐3, with h‐MSCs, and compared with 32D cell line co‐cultured on AML‐MSCs layer treated with Lubi (B, *n* = 5) or IVM (C, *n* = 5). *T*‐test was performed to compare treated groups or h‐MSCs versus AML‐MSCs, with ±IL3 used as experimental control. D) Total branches length of HUVEC tubes by using conditioned medium derived from h‐MSCs (*n* = 4) and AML‐MSCs untreated (*n* = 7) or pre‐treated with Lubi (*n* = 8) or IVM (*n* = 5) for 72 h and then stimulated (st) or not (unst) with a pro‐inflammatory cytokine cocktail for 24 h; tube formation was evaluated after 4 h and normalized to unst condition (AU: arbitrary unit, *t*‐test comparing treated or h‐MSCs stimulated groups versus stimulated AML‐MSCs). E) Percentage of PHA‐stimulated CD3^+^ *T*‐cells expressing CD69 and CD25 after 72 h of co‐culture on a layer of h‐MSCs or AML‐MSCs treated or not with Lubi (10 µm) or IVM (1 µm), relative to SF condition (*n* = 4, *t*‐test comparing treated or h‐MSCs groups versus AML‐MSCs). F,G) Relative expression of osteoprogenitor‐associated genes *TNAP* (F, *n* = 7 AML‐MSCs, *n* = 4 h‐MSCs) and *OPN* (G, *n* = 6 AML‐MSCs, *n* = 4 h‐MSCs) in h‐MSCs and AML‐MSCs treated or not for 72 h with Lubi (10 µm) or IVM (1 µm) and measured by RQ‐PCR (*t*‐test comparing treated or h‐MSCs groups versus AML‐MSCs). All histograms show mean ± SEM; ^*^ *p* < 0.05, ^**^ *p* < 0.01, ^***^ *p* < 0.001, ^****^ *p* < 0.0001.

Considering that h‐MSCs change their bioelectricity when leukemia occurs, we investigated whether depolarized AML‐MSCs could recover healthy hyperpolarized V_mem_ and healthy features. We hyperpolarized AML‐MSCs V_mem_ by using Lubi and IVM, and, after reducing V_mem_ (Figure 1J), we observed a lowered proliferation rate similar to h‐MSCs (IVM: −1.9‐fold, *p *< 0.05, **Figure** **4**A). Moreover, hyperpolarization reduced 32D cells support when cultured without IL‐3 (*p *< 0.01, Figure 4B,C), and recovered the anti‐inflammatory potential, confirmed by a greater anti‐angiogenic activity and higher potential to suppress *T*‐cell activation (*p *< 0.05, Figure 4D,E) at the same strength of h‐MSCs. Moreover, we observed a significantly reduced expression of osteo‐related genes *TNAP* (*p *< 0.0001, Figure 4F) and *OPN* (*p *< 0.05, Figure 4G), at recovering h‐MSCs level.

To definitively exclude a role of the drugs per se, we treated h‐MSCs with hyperpolarizing drugs (IVM, Lubi) and AML‐MSCs with depolarizing agents (K^+^ gluconate, Ouabain), showing that neither cell proliferation nor immunomodulatory functions were affected (Figure S4A,B, Supporting Information), thus excluding direct effects of the drugs on these functions.

### 2.5 CaV1.2 Expression is Linked to V_mem_

**Figure 5:**
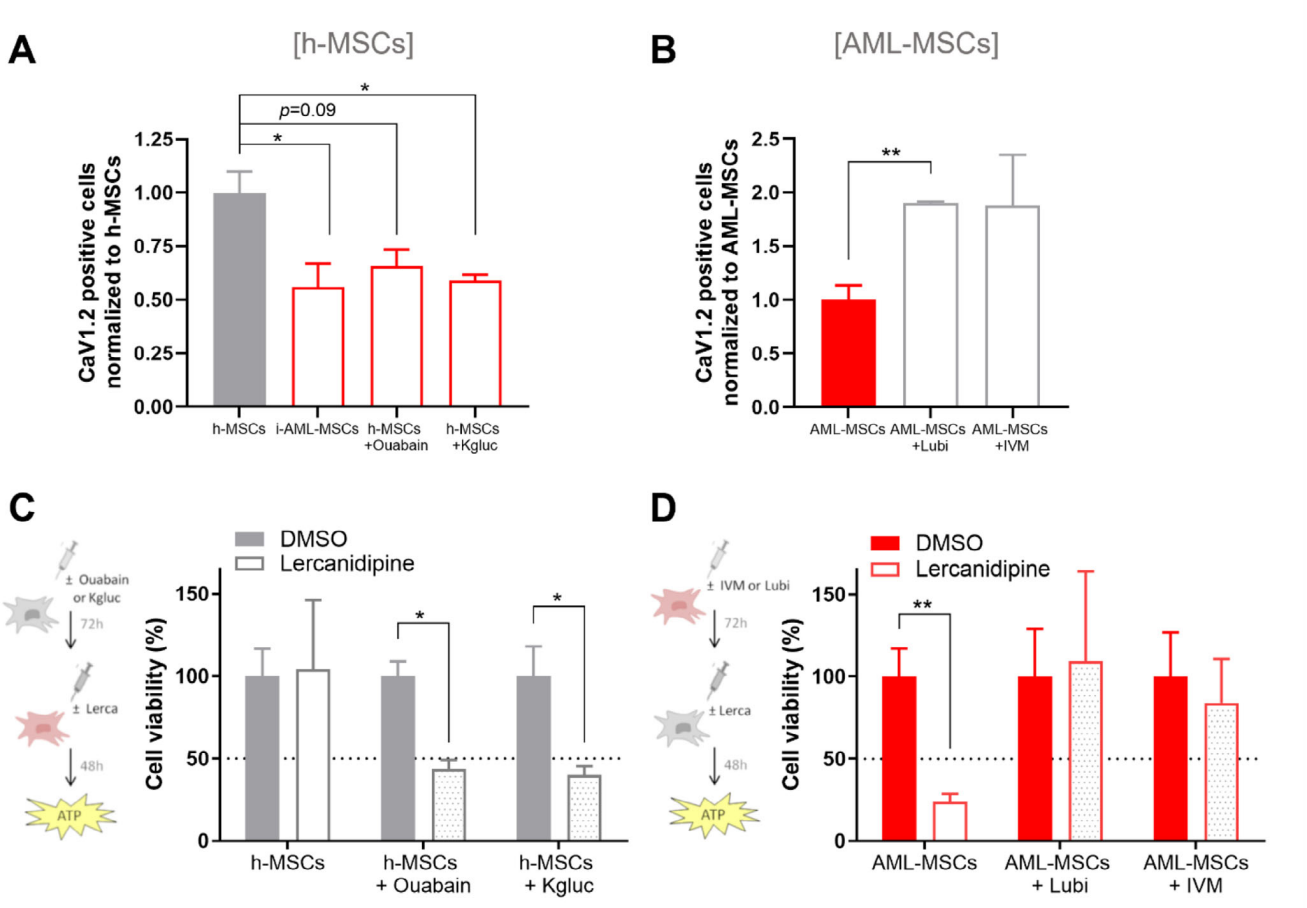
V_mem_ of MSCs controls CaV1.2 expression. A) CaV1.2 expression in h‐MSCs when co‐cultured with AML cells (red bar) for 4 days or treated with Ouabain or K^+^ gluconate (red bar) for 72 h, relative to h‐MSCs (grey bar), analyzed by flow cytometry (*n* = 3, *t*‐test comparing all groups versus h‐MSCs). B) CaV1.2 expression in AML‐MSCs when treated with Lubi or IVM (grey bar, *n* = 4) for 72 h, normalized to AML‐MSCs (red bar), analyzed by flow cytometry (t‐test comparing all groups versus AML‐MSCs). C) Cell viability was analyzed by ATP of h‐MSCs pre‐treated or not for 72 h with Ouabain or K^+^ gluconate and then incubated for 48 h with lercanidipine. ATP values were normalized to their respective untreated control (DMSO, *n* = 6, *t*‐test comparing all groups versus untreated h‐MSCs). D) Cell viability, measured by ATP, of AML‐MSCs pre‐treated or not for 72 h with Lubi or IVM and then for 48 h with lercanidipine, relative to DMSO value (*n* = 8, *t*‐test comparing all groups versus untreated AML‐MSCs). Dotted line represents 50% cell viability. All histograms show mean ± SEM; ^*^ *p* < 0.05, ^**^ *p* < 0.01.

Since we established a link among blasts, MSCs, and depolarization, we investigated whether Ca^2+^ and CaV1.2 channels were involved. We previously documented a different expression of CaV1.2 between h‐ and AML‐MSCs.^[^ ^26^ ^]^ Here, we showed that h‐MSCs reduced CaV1.2 protein expression when co‐cultured with AML blasts (<44%, *p *< 0.05, **Figure** **5**A; Figure S5, Supporting Information). We observed a similar down‐regulation of CaV1.2 expression in h‐MSCs depolarized for 72 h with Ouabain or K^+^ gluconate (<35%, *p *< 0.05, Figure 5A). In line with this finding, a 72 h treatment of AML‐MSCs with hyperpolarizing agents showed an up‐regulation of CaV1.2 protein expression (1.8‐fold, *p *< 0.01, Figure 5B; Figure S5, Supporting Information). To corroborate these findings, we blocked CaV1.2 with Lercanidipine, a selective CaV1.2 channel blocker previously shown to induce cell death in AML‐MSCs (expressing low CaV1.2 levels), whereas not altering Ca^2+^ influx nor viability of h‐MSCs (expressing higher levels of CaV1.2).^[^ ^26^ ^]^ In detail, we pharmacologically depolarized h‐MSCs or hyperpolarized AML‐MSCs, and after 72 h we treated cells with Lercanidipine, showing that h‐MSCs, when depolarized, became sensitive to Lercanidipine, reducing their viability similarly to AML‐MSCs (*p *< 0.05, Figure 5C); on the contrary, by hyperpolarizing AML‐MSCs, cells increased CaV1.2 expression and did not respond to Lercanidipine similarly to h‐MSCs (Figure 5D).

### 2.6 CaV1.2 Over‐Expression Reverts AML‐MSCs Phenocopying h‐MSCs Properties, Including Hematopoietic Stem Cells Support

**Figure 6:**
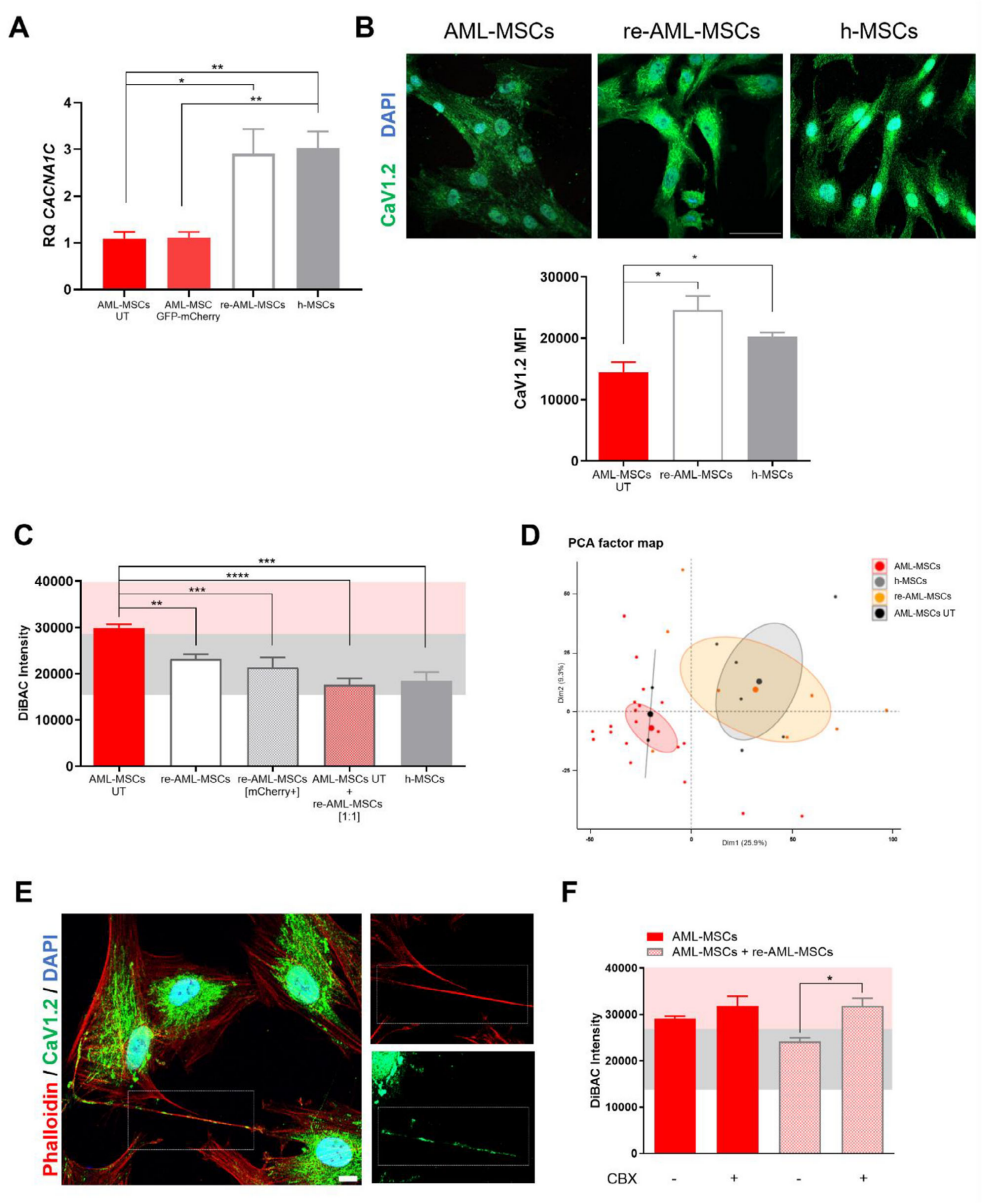
CaV1.2 expression controls MSCs V_mem_ depolarization. A) Relative *CACNA1C* (CaV1.2) mRNA expression measured by RQ‐PCR in AML‐MSCs UT, transduced with control GFP‐mCherry or CaV‐mCherry LVs (re‐AML‐MSCs, *n* = 11), or h‐MSCs (*n* = 3, *t*‐test comparing all groups versus AML‐MSCs). B) Representative images of immunofluorescence staining of CaV1.2 (green) and DAPI nuclear counterstain (blue) and relative quantification of CaV1.2 mean fluorescence intensity (MFI) (20X, 20 cells/image, 5 images, scale bar = 50 µm, *n* = 3 AML‐MSCs and re‐AML‐MSCs, *n* = 2 h‐MSCs, t‐test comparing all groups). C) DiBAC intensity of AML‐MSCs (*n* = 9), re‐AML‐MSCs total (*n* = 9) or gated on mCherry positive cells (*n* = 6), in a mixed culture of re‐AML‐MSCs and AML‐MSCs UT (ratio 1:1, *n* = 4), and h‐MSCs as control (*n* = 3). *T*‐test was adopted to compare all groups versus AML‐MSCs). D) Principal Component Analysis (PCA) performed on gene expression data of h‐MSCs (grey, *n* = 6), AML‐MSCs (red, *n* = 21), AML‐MSCs UT (black, *n* = 2), and re‐AML‐MSCs (orange, *n* = 8). Big dots represent the cluster centroid, and small dots represent samples. E) Representative images showing the presence of CaV1.2 channel inside the TNTs. Green indicates CaV1.2, blue indicates nuclei staining with DAPI, and red indicates F‐actin staining with Alexa647‐Phalloidin (100X, scale bar = 10 µm). F) DiBAC intensity in AML‐MSCs and in a mixed culture of re‐AML‐MSCs and AML‐MSCs UT (ratio 1:1), treated or not with 100 µm Carbenoxolone (CBX) for 72 h (*n* = 6). *T*‐test was applied to compare treated MSCs versus untreated. All histograms show mean ± SEM; ^*^ *p* < 0.05, ^**^ *p* < 0.01, ^***^ *p* < 0.001, ^****^ *p* < 0.0001.

Considering the lower CaV1.2 expression in AML‐MSCs for h‐MSCs, and the correlation of its levels with V_mem_, we hypothesized that recovering CaV1.2 expression in AML‐MSCs could correct their function. We first generated a plasmid containing CaV1.2 and tested its functionality on HEK293T, observing that CaV1.2 overexpression (Figure S6A, Supporting Information) led to an increased intracellular Ca^2+^ uptake in response to KCl depolarization (Figure S6B,C, Supporting Information). Next, we produced a CaV1.2 third‐generation lentiviral vector (LV) for a gene therapy approach in AML‐MSCs. Fourteen days post‐infection, we assessed CaV1.2 mRNA levels by RQ‐PCR, confirming its over‐expression in transduced AML‐MSCs, namely reverted AML‐MSCs (re‐AML‐MSCs) (*p *< 0.05, **Figure** **6**A), further confirmed by immunofluorescence staining of CaV1.2 (Figure 6B). Therefore, we measured re‐AML‐MSCs V_mem_ observing a more hyperpolarized status, reaching the DiBAC intensity of h‐MSCs (grey band, *p *< 0.001, Figure 6C). However, despite the transduction efficiency (mCherry^+^) ranging from 10% to 45%, we measured a more hyperpolarized V_mem_ also in mCherry‐negative cells with respect to un‐transduced (UT) MSCs, this latter finding arguing that the recovered hyperpolarized V_mem_ propagated to neighboring AML‐MSCs. To further elucidate this finding, we mixed AML‐MSCs UT with re‐AML‐MSCs (ratio 1:1) and demonstrated that V_mem_ was hyperpolarized in all cells and resembled that of h‐MSCs, supporting the hypothesis that re‐AML‐MSCs influenced the surrounding AML‐MSCs V_mem_ (Figure 6C). We performed gene expression profile analysis, and PCA showed that h‐ and re‐AML‐MSCs clustered together but separately from AML‐MSCs (Figure 6D), suggesting that CaV1.2 over‐expression triggered AML‐MSCs to hyperpolarization and to a healthy gene expression reprogramming. Moreover, interrogating the top up‐ and down‐expressed genes and the differentially expressed genes, we found that several known pathways (MAPK,^[^ ^29^ ^]^ PI3K,^[^ ^30^ ^]^ and NF‐κB^[^ ^31^ ^]^) potentially related to V_mem_ depolarization were deregulated in AML‐MSCs and that several genes were linked to niche remodeling (Tables S3 and S4, Supporting Information). We also investigated how re‐AML‐MSCs were able to propagate the depolarization wave to neighboring cells. We previously demonstrated in a 3D niche that MSCs contact local and distant cells by gap junctions and tunneling nanotubes (TNTs), thin F‐actin‐based membranous protrusions of MSCs, enabling the communication with the cytoplasm of neighboring cells^[^ ^26^ ^]^. We found that re‐AML‐MSCs formed TNTs with surrounding cells and that CaV1.2 localized and transited along these structures (Figure 6E). Moreover, treatment with CBX impaired the formation of TNTs between re‐AML‐MSCs and AML‐MSCs, significantly preventing the achievement of the bioelectric state of re‐AML‐MSCs in AML‐MSCs (Figure 6F).

**Figure 7:**
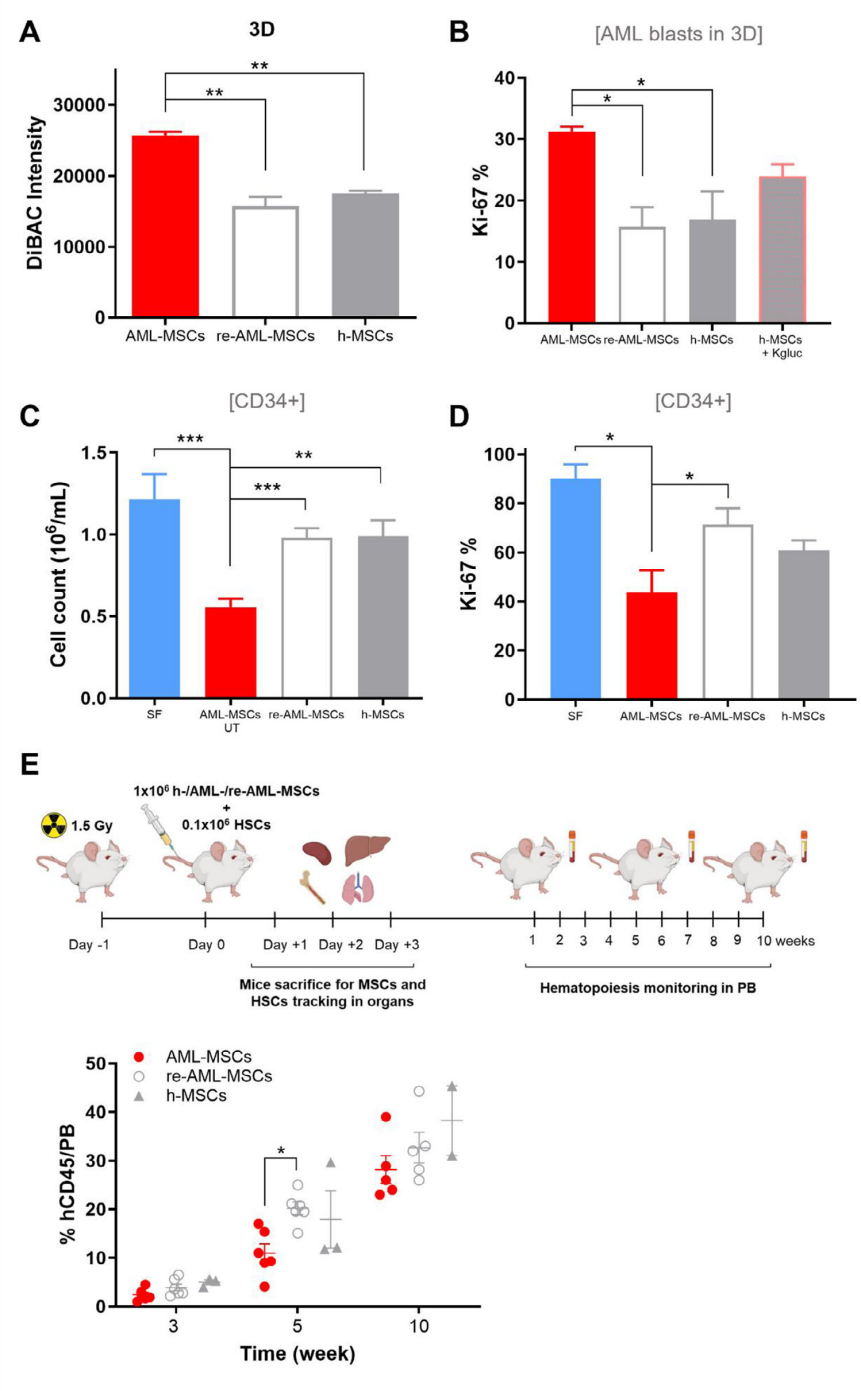
V_mem_ of MSCs influences leukemia proliferation and normal hematopoiesis. A) DiBAC fluorescence intensity of h‐MSCs (*n* = 2), AML‐MSCs (*n* = 3) and re‐AML‐MSCs (*n* = 2) in 3D. *T*‐test was performed to compare all groups. B) Percentage of Ki‐67 positive AML blasts after a 7‐day co‐culture in 3D with AML‐MSCs (*n* = 3), re‐AML‐MSCs (*n* = 2), h‐MSCs treated or not with K^+^ gluconate (*n* = 2, *t*‐test comparing all groups). C) Cell density of CD34^+^ cells after a 5‐day co‐culture with AML‐MSCs UT, re‐AML‐MSCs (*n* = 9), and h‐MSCs (*n* = 3). CD34^+^ cells cultured in SF conditions were used as controls. *T*‐test was performed to compare all groups. D) Percentage of Ki‐67 positive CD34^+^ cells after 5 days of co‐culture with AML‐MSCs UT, re‐AML‐MSCs, and h‐MSCs (*n* = 3, *t*‐test comparing all groups versus AML‐MSCs). E) On the top, schematic representation of the in vivo pipeline. On the bottom, percentage of human CD45 cells in NSG mice PB at 3, 5, and 10 weeks after tail vein co‐infusion of 1.0 × 10^5^ human cord blood CD34^+^ cells with 1.0 × 10^6^ h‐MSCs (*n* = 3 mice), AML‐MSCs (*n* = 6 mice), or re‐AML‐MSCs (*n* = 6 mice, *t*‐test comparing all groups). Created with BioRender.com. All histograms show mean ± SEM; ^*^ *p* < 0.05, ^**^ *p* < 0.01, ^***^ *p* < 0.001.

Considering that re‐AML‐MSCs are reprogrammed to h‐MSCs, we explored re‐AML‐MSCs functions. We documented that re‐AML‐MSCs reduced the growth of 32D cell line in the absence of IL‐3 with respect to AML‐MSCs (*p *< 0.05, Figure S7A, Supporting Information) and rescued immunosuppressive properties (Figure S7B, Supporting Information); however, proliferation and anti‐inflammatory potential of re‐AML‐MSCs were not significantly affected with respect to AML‐MSCs after 72 h (Figure S7C,D, Supporting Information). To further corroborate re‐AML‐MSCs reprogramming, we used our previously established 3D BM niche model^[^ ^26^ ^]^ and measured MSCs V_mem_ in a more complex in vitro system, confirming that AML‐MSCs were depolarized with respect to h‐MSCs and re‐AML‐MSCs, which were hyperpolarized (**Figure** **7**A). Therefore, considering the impact of AML‐MSCs in sustaining AML blasts (Figure S8A, Supporting Information), we evaluated blast proliferation in 3D when co‐cultured with different MSCs. Results showed a reduction of AML cells proliferation when co‐cultured with h‐MSCs or re‐AML‐MSCs with respect to AML‐MSCs or h‐MSCs depolarized with K^+^ gluconate (Figure 7B), this supports the direct role of MSCs in sustaining TME through V_mem_. Since h‐MSCs play a key role in controlling hematopoietic stem cells (HSCs) homeostasis and functions, we investigated re‐AML‐MSCs support to normal HSCs (CD34^+^). *CXCL12* and *ANGPT1* expression, critical for HSCs engraftment, homing, and proliferation,^[^ ^32^, ^33^ ^]^ was found remarkably up‐regulated in re‐AML‐MSCs compared with AML‐MSCs. *VEGFA*, which regulates HSCs quiescence,^[^ ^34^ ^]^ was significantly down‐regulated in re‐AML‐MSCs (*p *< 0.001, Figure S9A, Supporting Information). Hence, we asked whether the recovery of healthy features in re‐AML‐MSCs could restore their hematopoietic supportive capacity in vitro and in vivo. We compared the expansion of CD34^+^ cells cultured alone (SF) or on a layer of h‐, AML, or re‐AML‐MSCs, confirming that AML‐MSCs decreased HSCs proliferation (−2.2‐fold, *p *< 0.001, Figure 7C), whereas re‐AML‐MSCs and h‐MSCs supported CD34^+^ proliferation (*p *< 0.001, Figure 7C), also validated by Ki‐67 positivity (Figure 7D). In vivo, CD34^+^ HSCs were co‐infused by tail vein injection into sub‐lethally irradiated NOD/SCID/IL‐2Rγ null (NSG) mice together with h‐, AML‐, or re‐AML‐MSCs. For 10 weeks after transplantation, peripheral blood (PB) samples were analyzed for the presence of human CD45^+^ (hCD45^+^) cells. In line with in vitro data, we detected a significantly lower percentage of hCD45^+^ in the PB of NSG mice receiving CD34^+^ cells in co‐transplantation with AML‐MSCs as compared to re‐AML‐MSCs (*n* = 6 mice per group, *p *< 0.05, Figure 7E), as well as h‐MSCs. Since there are debated results on the role of MSCs in improving hematopoietic reconstitution,^[^ ^35^, ^36^ ^]^ we strengthened re‐AML‐MSCs capability of sustaining engraftment of CD34^+^ cells by using a second mice strain (humanized NOG‐EXL, Figure S9B, Supporting Information), confirming these findings. Thus, to link the increased CD34^+^ engraftment to the presence of MSCs, we monitored MSCs in vivo. In detail, we injected mCherry‐transduced MSCs in vivo and sacrificed mice at 24, 48, and 72 h post‐injection, demonstrating, in the window of CD34^+^ homing to murine BM, that MSCs were increasingly repopulating the BM, and migrated also in lung, spleen, and liver, as expected^[^ ^37^ ^]^ (Figure S9C, Supporting Information). Then, CD34^+^ cells were pre‐stained with CFSE to test their engraftment and proliferation in vivo when co‐injected with h‐, AML‐ or re‐AML‐MSCs. Results showed that re‐AML‐MSCs improved CD34^+^ cell proliferation with respect to AML‐MSCs (reduction of % CFSE^+^ cells in BM, Figure S10A,B, Supporting Information). These results documented that, for three days after injection, MSCs reside in the BM playing a direct role in contributing to HSCs engraftment and proliferation, thus sustaining hematopoiesis as demonstrated by hCD45^+^ cells in mice PB from 5 to 10 weeks post CD34^+^ and h‐ or re‐AML‐MSCs co‐injection (Figure 7E; Figure S9B, Supporting Information). These data highlight that MSCs co‐transplantation with donor's HSCs have the potential to increase an early graft, as previously reported.^[^ ^37^, ^38^, ^39^ ^]^ Overall, autologous re‐AML‐MSCs promote a successful early HSCs anchorage in the BM and contribute to their proliferation and to hematopoiesis recovery in vivo. Altogether, while in the context of disease onset we used lercanidipine to eradicate the pro‐leukemic stroma,^[^ ^26^ ^]^ in a transplant setting where the presence of healthy stromal cells facilitates donor HSCs engraftment, CaV1.2 levels restoration in MSCs could represent a promising strategy (Figure S11, Supporting Information).

## 3 Conclusion

Bioelectric signaling emerged in the last decades as an important controller of cell growth, cell migration, tumor progression, metastasis, and TME.^[^ ^11^ ^]^ It is nowadays accepted that aberrant bioelectrical interactions, mediated by altered ion channel expression and activity, can promote abnormal depolarization of resting potential and cell signaling also in non‐excitable cells.^[^ ^14^, ^20^ ^]^ MSCs are a biologically relevant component of the BM niche, being able to support hematopoiesis but also to differentiate along mesenchymal and non‐mesenchymal *lineages* to acquire immunomodulatory, reparative, and anti‐inflammatory properties.^[^ ^40^, ^41^, ^42^ ^]^ Recently, the therapeutic potential of MSCs has expanded beyond conventional applications, incorporating innovative cell‐based strategies such as engineered MSCs and CAR‐MSCs. These approaches aim to enhance the efficacy of cancer therapy by leveraging MSCs tumor‐homing properties and modulating the tumor microenvironment, broadening their role in targeted cellular therapies.^[^ ^43^, ^44^, ^45^, ^46^ ^]^ We previously characterized MSCs derived from AML patients, demonstrating that lercanidipine, a CaV1.2 channel blocker, was selective for AML‐MSCs, and impairs leukemia progression when combined with chemotherapy in the context of leukemia onset.^[^ ^26^ ^]^ We thus focused on CaV1.2, which is highly expressed in the brain and peripheral nervous system, regulating neuropathic pain,^[^ ^47^ ^]^ but its role in MSCs is largely unexplored. Here we found that Ca^2+^ dynamics were different in h‐ and AML‐MSCs, and questioned if tumor‐specific variations in ion balance contribute to confer leukemia aggressiveness, and induce the TME by affecting MSCs functions. We found that decreased density of CaV1.2 in AML‐MSCs mediated an aberrant Ca^2+^ uptake and membrane depolarization with respect to h‐MSCs, which are hyperpolarized, also confirmed in a 3D model of the BM niche. These findings indicate that AML‐MSCs help shape the leukemic niche through their electrical properties.^[^ ^8^, ^21^ ^]^ In line with this hypothesis, co‐culture experiments of h‐MSCs and AML blasts revealed that a stable MSCs depolarization occurred upon blasts exposure, reaching a depolarized V_mem_ comparable to AML‐MSCs. Particularly, MSCs retained a depolarized V_mem_ only after contact with blasts, whereas their V_mem_ returned to basal levels following PBMCs removal, providing evidence that AML blasts directly induce the irreversible MSCs bioelectric shift toward depolarization. So far, the underlying mechanism responsible for the propagation of the bioelectric state from tumor cells to neighbor stromal cells is largely underexplored. We previously observed that AML cells and AML‐MSCs formed networks through gap junctions in 3D.^[^ ^26^ ^]^ Here, we showed that the interruption of blast‐MSC communication, inhibiting or silencing Cx‐43, prevented the achievement of the depolarization status characterizing AML‐MSCs, demonstrating that AML blasts drive MSCs bioelectric and transcriptomic transition by cell–cell contact. Interestingly, in the leukemia context, recent reports showed that disruption of Cx‐43 on MSCs influences HSCs homeostasis and leukemia proliferation, as well as chemoresistance.^[^ ^48^, ^49^ ^]^

Since ion channels transmit extensive information patterns between different cell types and the environment, we hypothesized that tumor cells may not depend exclusively on altered genes for progression, but also on non‐autonomous cell interactions and ion balance. By pharmacological modulation of ion channels activity, we revealed that V_mem_ depolarization induced a pro‐leukemia‐like phenotype on h‐MSCs, supporting AML proliferation in a 3D model. Conversely, hyperpolarizing AML‐MSCs suppressed their pro‐leukemic phenotype, suggesting that V_mem_ hyperpolarization alone, independently of ion‐specific effects or the scaffolding roles of ion channel proteins, can modulate cell functions in the absence of other molecular interventions (such as gene knockout or RNA interference). Likewise, even if ion channel dysregulation in spatial or temporal tumor heterogeneity remains to be further explored, we lay the basis for considering bioelectricity as a potential feature influencing treatment efficacy, clonal evolution, and disease progression.^[^ ^50^ ^]^ In line with these findings, it was crucial to confirm the role of electrical signals in MSCs. We demonstrated that CaV1.2 over‐expression by lentiviral vectors in reverted AML‐MSCs (re‐AML‐MSCs) could, itself, repolarize AML‐MSCs toward a healthy hyperpolarized state, which propagated in a wave typical of excitable cells, and restored the h‐MSCs gene expression profile. Importantly, AML‐MSCs displayed gene and pathway deregulation that favored depolarization and sustained the leukemic niche, whereas h‐ and re‐AML‐MSCs exhibited a profile supporting normal hematopoiesis. We rationalized how electrical signals propagate and showed a nanotubes‐mediated CaV1.2 trafficking that caused V_mem_ hyperpolarization in neighboring cells. This latter finding of CaV1.2 hijacking from re‐AML‐MSCs to AML‐MSCs emerged as a novel mechanism to restore healthy MSCs. In addition, our gene therapy approach highlights the potential use of re‐AMLMSCs as a cellular product for their role in establishing a healthy BM niche. In fact, HSCs homing and engraftment still represent an important issue leading to graft failure. Several works revealed that the presence of a functional BM microenvironment capable of sustaining HSCs engraftment, expansion, and differentiation, and reducing graft‐versus‐host disease, is a fundamental requisite for a successful transplantation outcome.^[^ ^51^, ^52^, ^53^ ^]^ Here, to face an important therapeutic issue related to MSCs limited homing and long‐term persistence to target organs following administration, we tracked the presence of MSCs with HSCs in vivo three days after injection, observing a remarkable MSCs capacity to sustain HSCs homing and proliferation, confirming a direct role of MSCs in HSCs engraftment. Moreover, CaV1.2 restoration to autologous AML‐MSCs repolarized and restored Ca^2+^ dynamics and a healthy MSCs program. Despite the many questions that still need to be answered, these results of a gene therapy approach on MSCs in the context of HSCT has an enormous potential,^[^ ^35^, ^37^, ^38^, ^39^ ^]^ as supported by several different clinical trials recently emerged (NCT01045382, NCT06171906, NCT02291770, NCT02379442), including CAR‐MSCs which represent a widely applicable therapeutic technology in the field of immune‐therapy.^[^ ^43^ ^]^

In conclusion, our findings reveal a groundbreaking mechanism by which CaV1.2 expression modulates Ca^2^⁺ levels, influencing MSCs V_mem_ through a dynamic feedback loop dependent on cell–cell interactions. These discoveries unveil an unprecedentedly recognized bioelectric code governing TME in leukemia, with MSCs V_mem_ as a key regulatory feature in BM niche remodeling and hematopoietic support, thus marking a major step forward in understanding leukemogenesis. Overall, harnessing bioelectricity to restore homeostasis in leukemia could redefine future approaches in precision medicine, offering new hope for novel incoming cellular interventions.

## 4 Experimental Section

### Study Design

This study was designed to investigate the contribution of intracellular Ca^2+^ oscillation, observed in a 3D model of MSCs and leukemic blasts, in mediating primary MSCs V_mem_ changes (see Table S1, Supporting Information). Briefly, Ca^2+^ flux dynamics and V_mem_ changes in h‐MSCs and AML‐MSCs were measured (both derived from primary BM specimens) using the Ca^2+^ sensitive dye Fluo‐4, the voltage‐sensitive dye DiBAC_4_(3) (Bis‐(1,3‐diethylthiobarbituricacid) trimethine Oxonol) and patch clamp. Then, V_mem_ was pharmacologically modulated, and 32D cell line proliferation, HUVEC tube formation and lymphocytes activation, and CaV1.2 expression were tested in vitro. Primary AML‐MSCs were reverted (namely re‐AML‐MSCs) by over‐expressing CaV1.2 using lentiviral vectors and V_mem_ recovery, MSCs gene expression and functional properties, including hematopoietic stem cells (HSCs) sustainment and hematopoiesis support in vitro and in a transplant setting in vivo were studied.

### Ethics Approval and Consent to Participate

AML cells and derived MSCs were obtained from patients following AIEOP AML 2002/01 protocol. Written informed consent to utilize biological material from the leukemia cell characterization of children enrolled in the AIEOP AML 2002/01 protocol (EudraCT 2014‐000976‐25) was obtained by patients’ legal guardians, and the study was performed following the Declaration of Helsinki and our local institutional review board. In vivo experimental procedures were performed at the Venetian Oncologic Institute animal facility. Procedures involving animals were following institutional guidelines that comply with national and international laws and policies (EEC Council Directive 86/609, OJ L 358, 12 December 1987) and with “ARRIVE” guidelines (Animals in Research Reporting In Vivo Experiments). Italian Ministry authorization approval: 512/2019‐PR.

### MSCs Isolation, Culture, and Characterization

Isolation, culture, expansion, and characterization of MSCs from BM were conducted as previously published.^[^ ^26^ ^]^ Briefly, cells from BM of pediatric AML patients and healthy donors were plated at 100.000 cells cm^−2^ in StemMACS MSC Expansion Media (catalog number 130‐091‐680, Miltenyi Biotec, Bergisch Gladbach, Germany), supplemented with 100 U mL^−1^ penicillin/streptomycin (catalog number 15140122, Gibco, Thermo Fisher Scientific, Waltham, MA, United States of America) and incubated at 37 °C. After 1 day of culture, the non‐adherent cells were removed, and fresh medium was added. The resulting adherent cells were considered to be MSCs derived from AML at diagnosis (AML‐MSCS) or from healthy donors (h‐MSCs). These derived MSCs were characterized as per ISCT guidelines, confirming positivity for CD73 (clone JAD2, catalog number 12‐0739‐42, Invitrogen, Thermo Fisher Scientific), CD90 (clone 5E10, catalog number 561558, Beckman Coulter, Brea, CA, United States of America), and CD105 (clone 266, catalog number 560839, Becton Dickinson, Franklin Lakes, NJ, United States of America), and negativity for CD34 (clone 8G12, catalog number 34804, Becton Dickinson), CD45 (clone J33, catalog number A07784, Beckman Coulter), and CD11b (clone Bear1, catalog number IM2581U, Beckman Coulter), and their multipotency to differentiate into adipogenic, osteogenic, and chondrogenic lineages. All MSCs were expanded until 90% confluence and then re‐plated and expanded in larger flasks. At each passage, MSCs were trypsinized (Trypsin/EDTA Solution, catalog number 25200056, Gibco, Thermo Fisher Scientific) at 37 °C for 5′, harvested in medium with FBS (catalog number A5256701, Gibco, Thermo Fisher Scientific) to inactivate trypsin, spun and plated at 5 × 10^3^ cells/cm^2^ in fresh medium. All experiments were carried out using MSCs between passages 2 to 5.

### Cell Lines and Primary Cell Cultures

AML cell lines HL‐60 (catalog number ACC 3) and SHI‐1 (catalog number ACC 645, DSMZ, Braunschweig, Germany), primary AML blasts, and Human Umbilical Vein Endothelial Cells (HUVEC, catalog number C2519A, Lonza, Basel, Switzerland) were cultured as previously described.^[^ ^26^ ^]^

Healthy CD34^+^ cells were isolated from human cord blood (CB) mononuclear cells (PBMCs) obtained by centrifugation over Lymphoprep (catalog number 1856, Serumwerk Bermbrug AG, Bernburg, Germany), followed by incubation with CD34 MicroBeads (catalog number 130‐046‐702, Miltenyi Biotec). CD34^+^ cells were isolated by magnetic cell sorting and cultured in MyeloCult H5100 medium (catalog number 05150, Stem Cell Technologies, Vancouver, Canada), supplemented with cytokines (100 ng mL^−1^ hSCF catalog number 130‐096‐692, and hFlt3L catalog number 130‐096‐474; 20 ng mL^−1^ hIL‐3 catalog number 130‐093‐909, hIL‐6 catalog number 130‐093‐929, and hG‐CSF catalog number 130‐096‐346, Miltenyi Biotec).

HEK293T (catalog number ACC 635, DSMZ) cells were maintained in DMEM supplemented with 10% FBS (Thermo Fisher Scientific, Waltham, MA, United States of America), 2 mm glutamine (catalog number 25030081, Gibco, Thermo Fisher Scientific), and 100 U mL^−1^ streptomycin/penicillin (Gibco, Thermo Fisher Scientific).

### Ca2+ Flux Imaging

Ca^2+^ flux was monitored by fluorescence imaging using the Ca^2+^ sensitive dye Fluo‐4 (catalog number F14201, Invitrogen, Thermo Fisher Scientific) as previously described.^[^ ^26^ ^]^ Time‐lapse series were taken at 2 s intervals over a period of 40–400 seconds to capture Ca^2+^ oscillations.

FIJI ImageJ software was used for image processing, including drawing of regions of interest around individual cells and calculating average pixel intensity per cell per time. Fluorescence intensity was normalized to minimum intensity values and monitored over time for each cell.

For amplitude calculation, maximal (F_max_) and minimal (F_min_) fluorescence values were determined, and data were presented as the relative change in fluorescence. After monitoring baseline oscillations, Ca^2+^ levels were determined before and during the exogenous administration of KCl solution (65 mm) and then imaged for an additional 50–60 min. Intracellular Ca^2+^ levels were also determined after treatment with 10 nm Ouabain (catalog number PHR1945, Sigma‐Aldrich, Merck, Darmstadt, Germany) for 72 h.

### Vmem Measurements by DiBAC Staining

To measure V_mem_ changes at the predetermined timepoints during cell proliferation and drug treatment, MSCs were loaded with the voltage‐sensitive dye Bis‐(1,3‐diethylthiobarbituricacid) trimethine Oxonol (DiBAC_4_(3), catalog number B438, Invitrogen, Thermo Fisher Scientific). The extent of DiBAC binding is inversely proportional to the magnitude of the voltage across the membrane; thus, its uptake is voltage‐dependent and higher in depolarized cells. MSCs were seeded at a density of 2.5 × 10^3^ cells cm^−2^ in a 24‐well glass‐bottom plate (Ibidi GmbH, Gräfelfing, Germany). After 72 h, a fresh solution of 1 mm DiBAC_4_(3) in DMSO was prepared and diluted to 1 µm in Hank's Buffered Salt Solution (catalog number 3‐02F33‐I, BioConcept Ltd, Allschwil, Switzerland). After dye addition, cells were incubated for 20 min at 37 °C, then imaged using Zeiss LSM800 Airyscan Confocal microscope (Zeiss, Oberkochen, Germany). The DiBAC_4_(3) dye was excited with a 488 nm light, and fluorescence was captured at 510/530 nm. Since fluorescence intensity was quantified for each image, gain, exposure time, and offset settings of the microscope were kept constant throughout each experiment. Fluorescence intensity was quantified by the FIJI ImageJ software.

To evaluate the influence of external Ca^2+^ mobilization on V_mem_ modulation, MSCs were treated with the extracellular calcium chelator EGTA (2 mm, catalog number 324628, Sigma–Aldrich, Merck), whereas the effect of intracellular calcium was studied by using the calcium chelator BAPTA‐AM (50 µm, catalog number S7534, Selleckchem, Houston, TX, United States of America).

### Intracellular Vmem Recordings Through Perforated Patch Clamp

V_mem_ was recorded in AML‐MSCs and h‐MSCs cultured either alone or with AML blasts after 24–72 h from seeding. Cells were resuspended over a glass previously coated with fibronectin 40 µg mL^−1^ (Corning Life Sciences, Glendale, AZ, United States of America) to a chamber at room temperature (RT). The extracellular solution contained 150 mm NaCl, 5 mm KCl, 2 mm CaCl_2_, 1 mm MgCl_2_, 2 mm sodium pyruvate, 10 mm HEPES, 5 mm glucose with 310 Osm and pH adjusted to 7.4 using NaOH. DiBAC 1 µm was added while waiting 15 min for the cells to precipitate on the coverslip. Borosilicate glass capillary tubing (1.5 mm OD, Warner Instrument Corp., Hamden, CT, United States of America) was used, pulled with a vertical puller (Model P‐830, Narishige International, Japan), resulting in pipette resistances in the range of 6–8 MΩ. Pipettes were filled with an intracellular solution containing 115 mm potassium aspartate, 10 mm NaCl, 10 mm KCl, 1 mm MgCl_2_, 10 mm HEPES, 4 mm BAPTA with 285 Osm and pH adjusted to 7.2 with KOH. Gramicidin A 0.1 mg mL^−1^ (catalog number 50845, Sigma–Aldrich, Merck) was added freshly each day of recordings.

Cell currents were amplified by a Multiclamp 700b amplifier (Molecular Devices, CA, United States of America) and recorded by the pClamp software (version 10.4, Molecular Device) after digitization at 10 KHz by a Digidata 1550A (Molecular Devices). Data analysis was performed by custom‐made software in MatLab 2019b (MathWorks Inc., MA, United States of America). DiBAC fluorescence was excited by a 470 nm collimated LED (Thorlabs, NJ, United States of America), and CD45 fluorescence with a 565 nm collimated LED (Thorlabs) filtered with 535/50 BP and 610/LP filters, respectively. The fluorescence light was collected using an upright wide‐field microscope (BX51WI, Olympus, Tokyo, Japan) equipped with an infinity‐corrected water immersion objective (40x, 0.8 NA, Olympus) and a sCMOS cooled camera (PCO.edge 5.5) at 0.1 Hz frame rate.

To achieve perforation, the patched cell was held at 0 mV in cell‐attached configuration until Gramicidin A was allowed to record intracellularly, normally after 10 to 15 min. DiBAC initial fluorescence was then measured (F_0_) before switching to current clamp at 0 pA in order to measure the resting V_mem_. In this configuration, DiBAC fluorescence (F) was measured once stabilized for at least 5 min, and then holding again the cell at 0 mV to check for F_0_ reliability. DiBAC relative fluorescence variation, ΔF/F_0_ = (F‐F_0_)/F_0_, was found to be proportional to V_mem_ in first approximation.

### Pharmacological Vmem Modulation

Several drugs were employed to modulate V_mem_ in MSCs: 1) Na^+^/K^+^‐ATPase‐inhibitor Ouabain (5–25 nm, Sigma‐Aldrich, Merck) was added to the medium from a fresh 10 mm stock solution in DMSO; 2) the concentration of extracellular K^+^ was increased by adding potassium gluconate (K^+^ gluconate, catalog number G4500, Sigma‐Aldrich, Merck) to the medium to final concentrations of 10–80 mm; 3) the chloride channel (ClC‐2) activators Lubiprostone (Lubi, catalog number S1675, Selleckchem) and Ivermectin (IVM, catalog number PHR1380, Sigma‐Aldrich, Merck) were added to the medium to final concentrations of 0.1–1 and 1–50 µm, respectively, from 10 mm stock solutions in DMSO. For all experiments, MSCs were seeded at 5 × 10^3 ^cells cm^−2^ in StemMACS MSC Expansion Media (Miltenyi Biotec); the day after, cells were treated with the depolarizing/hyperpolarizing agent for 72 h. In order to evaluate the reversibility of the effect, a drug washout procedure was performed, and after 72 h its effects were evaluated.

To explore the influence of V_mem_ in CaV1.2 modulation, MSCs pre‐treated with depolarizing/hyperpolarizing agents for 72 h were then exposed to the CaV1.2 blocker lercanidipine (10 µm, catalog number L6668, Sigma‐Aldrich, Merck) for 48 h.

### Cell Proliferation Assay

The MSCs proliferation was evaluated using PrestoBlue Cell Viability Reagent (catalog number A13261, Invitrogen, Thermo Fisher Scientific) following the manufacturer's guidelines. Briefly, 2,5 × 10^3^ MSCs were cultured in 100 µL of proper medium in sterile 96‐well plates and incubated at 37 °C, then at the indicated timepoints, 10 µL of PrestoBlue reagent was added. Plates were incubated for 1 h at 37 °C, and absorbance was recorded using a Spark Multimode Microplate Reader (TECAN, Männedorf, Switzerland) at 570 nm with a reference wavelength of 600 nm.

### MSCs‐32D Co‐Culture and Differentiation Evaluation

IL‐3‐dependent 32D murine hematopoietic precursor cells (catalog number ACC 411, DSMZ) were cultured for 72 h on a layer of depolarized or hyperpolarized MSCs, as previously described.^[^ ^26^ ^]^

To assess 32D cell differentiation, 32D cells in suspension were collected and centrifuged to allow attachment to the slice, then stained by May–Grünwald Giemsa method.

### HUVEC Tube Formation Assay with Conditioned Medium of MSCs

HUVEC Tube Formation Assay was conducted as published previously,^[^ ^26^ ^]^ by using conditioned media derived from depolarized and hyperpolarized‐MSCs.

### Lymphocytes Activation Assay

Anti‐inflammatory potential of depolarized‐ and hyperpolarized‐MSCs was tested, evaluating the activation of proliferating lymphocytes, as previously described.^[^ ^26^ ^]^

### Human IL‐6 ELISA Assay

IL‐6 protein levels in culture supernatants were measured using ELISA (catalog number KHC0061, Thermo Fisher Scientific), following manufacturer's instructions. Absorbance A450 values were measured using a microplate reader (Spark, TECAN).

### RNA Isolation and Quantitative Real‐Time PCR

Total RNA was isolated using Trizol (catalog number 15596026, Invitrogen, Thermo Fisher Scientific). One µg of RNA was reverse‐transcribed into cDNA using the SuperScript II system (catalog number 18064014, Invitrogen, Thermo Fisher Scientific) according to the manufacturer's instructions. Expression of mRNA was measured by Real Time PCR (RQ‐PCR) on an ABI 7900HD platform (Applied Biosystems, Thermo Fisher Scientific) using the SYBR Green PCR master mix (catalog number 11733046, Applied Biosystems, Thermo Fisher Scientific) and normalized on *GUSB* housekeeping gene, using the 2^−∆∆Ct^ method. Primers sequences for the selected genes are reported in **Table** **1**.

**Table 1:** Sequence of primers used throughout the manuscript.

Gene	Forward	Reverse
*OPN*	TTGCAGCCTTCTCAGCCAA	GGAGGCAAAAGCAAATCACTG
*TNAP*	CCTCCTCGGAAGACACTCTG	GCAGTGAAGGGCTTCTTGTC
*IL‐6*	CTGCAGCCACTGGTTCTGT	GATGAGTACAAAAGTCCTGATCCA
*PTGS2*	GTTCCACCCGCAGTACAGAA	AGGGCTTCAGCATAAAGCGT
*CACNA1C*	GGTCAACTCCACCTACTTCGAGT	GCATCACAGAAATAGTGCTTGGGTT
*CXCL12*	ATGAACGCCAAGGTCG	GGGCTACAATCTGAAGGG
*ANGPT1*	GTTCAGAACCACACGGCTACC	CCAGCAGCTGTATCTCAAGTCG
*VEGF*	GCCTCCGAAACCATGAACTTT	TGGTGGAGGTAGAGCAGCAA
*CX43*	AGCCACTAGCCATTGTGGAC	CCACCTCCACCGGATCAAAA
*GUS*	GAAAATATGTGGTTGGAGAGCTCATT	CCGAGTGAAGATCCCCTTTTTA

### AML‐Induced MSCs Co‐Culture Model

h‐MSCs were plated at 5 × 10^3^ cm^−2^ and, after 24 h, co‐cultured with AML primary blasts or AML cell lines (ratio 1:10) for a time between 30 min and 7 days in AML cells proper medium. After being co‐cultured with AML cells, h‐MSCs were named “induced AML‐MSCs” (iAML‐MSCs). As control, h‐MSCs were co‐cultured with CD34^+^ cells from CB.

The role of cell–cell contact was investigated by silencing Connexin‐43 (Cx‐43, catalog number 4392420, assay ID 144485and 144486, Ambion, Thermo Fisher Scientific) in AML blasts and h‐MSCs or by treatment with Carbenoxolone (CBX, 100 µm, catalog number C4790, Sigma‐Aldrich, Merck) for 4 days of co‐culture. The role of AML blasts secretome was evaluated by using a 0.4 µm polycarbonate Transwell insert (Corning Life Sciences) to separate AML blasts from MSCs for 4 days.

### siRNA Transfection in Primary Cells

Cx‐43 was silenced in AML primary blasts and h‐MSCs, using electroporation or calcium phosphate methods as previously described.^[^ ^26^ ^]^

### Intracellular Staining for Flow Cytometry Analysis

Cells were fixed in 4% formaldehyde for 10 min, then permeabilized in 0.1% Tween20 (catalog number P1379, Sigma‐Aldrich, Merck) for 20 min, blocked with 3% bovine serum albumin (BSA, catalog number A7030, Sigma‐Aldrich, Merck) for 30 min, and incubated with 1:500 primary anti‐human CaV1.2 antibody (catalog number ACC‐003, Alomone labs, Jerusalem, Israel) in 3% BSA+20% FcR Blocking Reagent (catalog number 130‐059‐901, Miltenyi Biotec) for 30 min. Next, cells were incubated with Alexa Fluor 488 Goat Anti‐Rabbit IgG (H+L) secondary antibody (1:500, catalog number A32731, Thermo Fisher Scientific) for 30 min at RT, washed, and analyzed with CytoFLEX (Beckman Coulter).

### Transient Plasmid Transfection of HEK293T

Cells were grown as a monolayer to confluence up to 70% in a 35 mm Petri dish before transfection. Transient transfection was performed using the calcium phosphate method, using 0.33 µg of plasmid encoding CaV1.2‐mCherry (*pLV[Exp]‐mCherry‐hPGK>hCACNA1C*, Vector Builder Inc., IL, United States of America).

### MSCs Transduction with Lentiviral Vectors

MSCs at passage 1 were seeded at a density of 1.5 × 10^5^ cells cm^−2^ in 6‐well plates in a final volume of 2 mL. The day after, the vial containing the LV (Vector Builder Inc.) was thawed and added to MSCs with protamine sulfate (100 µg mL^−1^ in DMEM, catalog number P3369, Sigma‐Aldrich, Merck), at a multiplicity of infection (MOI) of 50. The volume of LV used was calculated as follows: MOI x N/TU, where TU is the amount of functional LV particles per unit of volume, and N is the number of cells. The plate was incubated at 37 °C for 18 h and then cells were extensively washed.

MSCs were transduced in parallel with CaV1.2‐mCherry (*pLV[Exp]‐mCherry‐hPGK>hCACNA1C*) and control GFP‐mCherry (*pLV[Exp]‐EGFP:T2A:Puro‐EF1A>mCherry*, Vector Builder) LVs. The efficiency of transduction was measured after 7 and 14 days by detecting the mCherry positivity by flow cytometry on the BD FACSCelesta (BD). CaV1.2 over‐expression was verified by RQ‐PCR and by flow cytometry after 14 days from transduction. Un‐transduced (UT) MSCs, incubated only with protamine sulfate, were used as control.

### Immunofluorescence

Cells were fixed in 4% formaldehyde for 10 min, washed in PBS 1X, permeabilized with 0.5% Triton X‐100 (catalog number X100, Sigma–Aldrich, Merck) for 20 min, washed in PBS 1X, blocked with 1% BSA (Sigma‐Aldrich, Merck). After 30 min, cells were subjected to immunofluorescence using the anti‐CaV1.2 antibody (1:200, Alomone labs) overnight at 4 °C. Primary antibody incubation was followed by incubation with Alexa Fluor 488 Goat Anti‐Rabbit IgG (H+L) secondary antibody (Thermo Fisher Scientific) for 2 h at RT. Cells were counterstained with 4ʹ,6‐diamidino‐2‐phenylindole (DAPI, 1:10000, catalog number D9542, Sigma‐Aldrich, Merck) to label nuclei and with Alexa Fluor 647 Phalloidin (catalog number A22287, Invitrogen, Thermo Fisher Scientific) to label F‐actin. Images were acquired using Zeiss LSM800 Airyscan Confocal microscope (Zeiss). Fluorescent intensity of MSCs was quantified by FIJI ImageJ software. Data were represented as mean pixel intensity ± SEM.

### Gene Expression Analysis of MSCs

RNA collected from 6 h‐MSCs, 21 AML‐MSCs, 2 AML‐MSCs UT, and 8 re‐AML‐MSCs after 3 passages of in vitro culture were analyzed. RNA quality was assessed on an Agilent2100 Bioanalyzer (Agilent Technologies, Santa Clara, CA, United States of America). Then, 100 ng of total RNA were labeled and hybridized to GeneChip Human Genome U133 Plus 2.0 Array (catalog number 900466, Applied Biosystems, Thermo Fisher Scientific) for 16 h at 45 °C using a rotational oven and washed according to Affymetrix standard protocols using a GC450 Fluidics Station (Applied Biosystems, Thermo Fisher Scientific). The GeneChip were scanned with an Affymetrix 7G scanner and CEL files generated were normalized by robust multi‐array averaging (RMA) algorithm with Affy‐R package (www.r‐project.com). ComBat function in SVA package was used to eliminate batch effect among experiments. Principal Component Analysis (PCA) using probe sets with more than 90% of variance variability was obtained.

To underpin pathways deregulated in AML‐MSCs+AML‐MSCs UT (*n* = 21 and *n* = 2 respectively) versus h‐MSCs+re‐AML‐MSCs (*n* = 6 and *n* = 8 respectively) samples, differentially expressed genes (*p* < 0.05) were considered and analyzed with the Enrichr tool (https://maayanlab.cloud/Enrichr/) to investigate Gene Ontology‐Biological Processes.

The datasets generated and analyzed during the current study were deposited in the GEO repository, with the access code GSE248681.

### 3D Culture System Setup

The 3D bone marrow niche model was based on the use of a biomimetic scaffold composed of hydroxyapatite and collagen type I. Scaffolds seeding with primary MSCs and AML cells was conducted as previously reported.^[^ ^26^ ^]^

### Vmem Analysis in 3D

To measure V_mem_ in 3D after one week of culture, the voltage‐sensitive dye DiBAC_4_(3) was diluted to 1 µm and added to the culture media for 20 min at 37 °C. To stain for nuclei, Hoechst 33342 (catalog number 62249, Thermo Fisher Scientific) at 8 µm was subsequently added for 10 min at 37 °C. After loading, scaffolds were immediately imaged using Zeiss LSM800 Airyscan Confocal microscope (Zeiss). Fluorescence intensity was quantified by FIJI ImageJ software.

### 3D‐AML Culture Staining

The 3D culture was fixed with 4% Paraformaldehyde for 30′ at 4 °C, then cells were permeabilized in situ with 0.5% TritonX‐100 (Sigma‐Aldrich, Merck) and 5% BSA (Sigma‐Aldrich, Merck) in PBS 1x for 1 hour at RT. For immunofluorescence staining, the primary antibody (Ki‐67 1:200, catalog number GA626, Dako, Agilent Technologies) was diluted in 0.1% TritonX‐100 and 1% BSA in PBS 1x and incubated for 24 h at RT in agitation. For immunofluorescence detection, the samples were incubated with Alexa488‐conjugated secondary antibody (Thermo Fisher Scientific, 1:200 in PBS 1x with 1% BSA) overnight at RT. F‐actin and nuclei were labeled with Alexa Fluor 647 Phalloidin (1:500, Invitrogen, Thermo Fisher Scientific) and 4′,6‐diamidino‐2‐phelylidine dihychloride (DAPI Sigma‐Aldrich, Merck, 1:1000) respectively, in PBS 1x, for 3 h at RT. After 3 washes the samples were transferred on a bottom glass chamber slides (Ibidi) for confocal imaging using the Zeiss LSM800 Airyscan Confocal microscope (Zeiss).

### MSCs and Healthy CD34+ Co‐Culture

MSCs and CD34^+^ isolated from CB of healthy donors were co‐cultured as previously described.^[^ ^26^ ^]^ 1 × 10^5^ CD34^+^ were plated in 500 µL of MSCs conditioned medium on a MSCs layer. After 5 days, CD34^+^ were collected and counted using Trypan Blue. The percentage of high proliferative index was evaluated by flow cytometry analysis, after incubating with Ki‐67‐488 (catalog number 53‐5698‐82, Invitrogen, Thermo Fisher Scientific) for 10 min at RT in the dark, using CytoFLEX (Beckman Coulter). CD34^+^ cells alone (stroma‐free, SF) or on a layer of h‐MSCs in HSCs proper medium were used as controls.

### Transplantation of Human CB CD34+ Cells and MSCs in Mice

Adult NSG (7 weeks old) or NOG‐EXL (NOD.Cg‐Prkdc^scid^ Il2rg^1Sug^ Tg(SV40/HTLV‐IL3, CSF2)10‐7Jic/JicTac, catalog number 13395‐F, Taconic Biosciences, Germantown, NY, United States of America) female mice were sub‐lethally irradiated (1.5 Gy) and transplanted via tail vein injection with 1 × 10^5^ human CD34^+^ cells alone or in combination with 1 × 10^6^ human MSCs. Mice were housed in individually ventilated cages, with bedding changed at least once a week, in a light‐dark cycle with controlled temperature and humidity and unlimited access to food and water. Animal suffering was minimized, and mouse welfare continuously monitored. Starting from 21 days post co‐infusion, peripheral blood (PB) samples were collected weekly from sub‐mandibular vein. The presence of human cells was determined by flow cytometry using mouse CD45 (clone 30‐F111, catalog number 552848, Becton Dickinson) and human CD45 (clone J33, catalog number A07784, Beckman Coulter) antibodies. All samples were run on a CytoFLEX (Beckman Coulter), and at least 10.000 events were recorded.

### CFSE Staining in CD34+ Cells

For the cell proliferation assay, 1 × 10^6^ CD34+ cells were resuspended in 1 mL of RPMI 1640 (no supplements added, catalog number 5240025, Gibco, Thermo Fisher Scientific) with the addition of CellTrace Violet Cell Proliferation Kit at the final concentration of 5 µm (catalog number C34557, Life Technologies, Thermo Fisher Scientific). After a 20 min incubation at 37 °C, 5 mL of RPMI 1640 (supplemented with 10% FBS, 2 mm glutamine, and 100 U mL^−1^ streptomycin/penicillin) was added, and cells were incubated for five additional minutes at 37 °C, then washed and injected into mice.

### In Vivo Tracking of MSCs and CD34+ Cells

Adult NOG‐EXL (7 weeks old) female mice were sub‐lethally irradiated (1.5 Gy) and transplanted via tail vein injection with 1 × 10^5^ human CD34^+^ cells CFSE‐stained with 1 × 10^6^ human MSCs transduced to express the mCherry reporter gene for MSCs tracking. Mice were sacrificed at 24, 48, and 72 h post‐injection to evaluate the presence of MSCs in the different mice organs. Following ethical guidelines, animals were euthanized by cervical dislocation. At 72 h, CD34+ cell proliferation was evaluated in murine bone marrow. Cells were monitored for fluorescence by flow cytometry on the BD FACSCelesta (BD) cytometer (Becton Dickinson). Data were analyzed using FlowJo v10 software (FlowJo LLC, Becton Dickinson).

### Statistics

Statistical analyses were performed using Prism6 (Graph Pad Software Inc., La Jolla, CA, United States of America). All experiments were performed at least in duplicate with specific numbers detailed in Figure legends and Table S1 (Supporting Information). When needed, data were normalized to the control group by calculating the mean of the control replicates and expressing each value (from both control and experimental groups) as a ratio relative to this mean. In all graphs, results were presented as mean ± SEM. Statistical significance of differences between two groups was evaluated by applying the Student's *t*‐test (two‐sided), after a preliminary testing of normal data distribution using the Shapiro–Wilk test. When comparing more than two groups, the Holm‐Šídák correction was applied for multiple statistical hypothesis testing. Results were considered statistically significant when *p*‐value was <0.05 (*), <0.01 (**), <0.001 (***), and <0.0001 (****).

## Author Contributions

Ambra Da Ros, Maddalena Benetton, and Giulia Borella contributed equally to this work., G.B., M.B., A.D.R., and M.P. contributed to conceptualization. G.B., M.B., A.D.R., G.L., G.B., A.C., D.L.P., C.T., M.B., S.B., and M.P. contributed to methodology. G.B., M.B., A.D.R., D.L.P., and S.B. contributed to validation. G.B., M.B., A.D.R., G.B., D.L.P., and S.B. contributed to formal analysis. G.B., M.B., A.D.R., G.L., G.B., A.C., D.L.P., and C.T. contributed to the investigation. G.B., M.B., A.D.R., G.B., D.L.P., M.B., S.B., and M.P. were involved in data curation, and S.B. performed software analyses. G.B., M.B., and A.D.R. contributed to visualization. M.B., F.L., and M.P. provided study samples and materials. G.B., M.B., A.D.R., and M.P. wrote the original draft, and G.B., M.B., A.D.R., C.T., F.L., and M.P. reviewed and edited the manuscript. C.T. and M.P. administered the project, supervised by F.L. and M.P. M.P. acquired funding to support this study. All authors read and approved the final manuscript.

## Conflict of Interest

The authors declare no competing interests.

## Supporting information



Supporting Information

Supporting Information

## Data Availability

The data that support the findings of this study are available from the corresponding author upon reasonable request. The datasets generated during the current study are available in the GEO repository, with the access code GSE248681. To perform the analyses reported in this study, data were analyzed together with those previously deposited at GSE169428 (https://doi.org/10.1182/blood.2020009845).
