# Supplementary material for: Leukemic Cells Hijack Stromal Bioelectricity to Reprogram the Bone Marrow Niche via CaV1.2‐Dependent Mechanisms
Source: Adv Sci (Weinh). 2025 Aug 20;12(39):e08940. doi: 10.1002/advs.202508940 (PMC12533209; doi:10.1002/advs.202508940)
Supplement: Supplementary file 1 — Supporting Information [file ADVS-12-e08940-s001.pdf]

ADVANCED SCIENCE
Open Access

Supporting Information

for *Adv. Sci.*, DOI 10.1002/advs.202508940

Leukemic Cells Hijack Stromal Bioelectricity to Reprogram the Bone Marrow Niche via CaV1.2-Dependent Mechanisms

*Ambra Da Ros, Maddalena Benetton, Giulia Borella, Giorgia Longo, Giulia Borile, Alice Cani, Diego Lopez-Pigozzi, Mario Bortolozzi, Silvia Bresolin, Claudia Tregnago, Franco Locatelli and Martina Pigazzi**

Leukemic Cells Hijack Stromal Bioelectricity to Reprogram the Bone Marrow Niche via CaV1.2-Dependent Mechanisms

Da Ros A. *et al.*

SUPPLEMENTARY FIGURES.

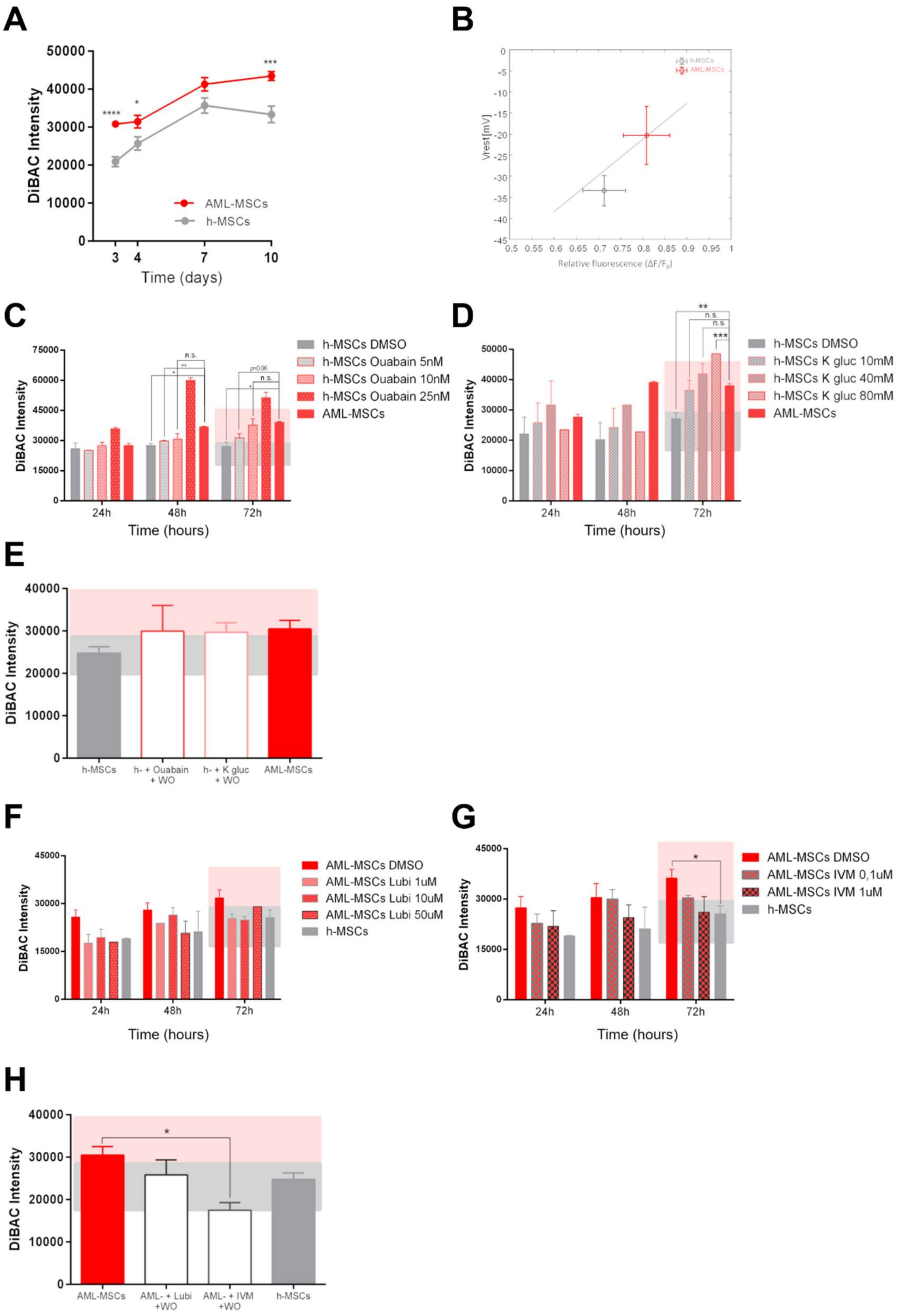

Supplementary Figure S1. MSCs V_{mem} and its modulation by drugs. (A) Time course of fluorescence intensity calculated from DiBAC fluorescence staining in AML-MSCs (n=14) and h-MSCs (n=7) 3, 4, 7 and 10 days after seeding. Graph shows the mean ± SEM. (B) Linear regression model between mean relative fluorescence intensity ($\Delta F/F_0$) of DiBAC and Vrest (mV) values derived from patch clamp calculated for each group (R^2=0.69, n=5 AML-MSCs, n=4 h-MSCs). (C) Dose-time curve of DiBAC fluorescence intensity in h-MSCs treated with Ouabain at 5, 10, 25 nM for 72 hours (n=5). AML-MSCs were used as control (n=3). (D) Dose-time curve of DiBAC fluorescence intensity in h-MSCs treated with K^+ gluconate at 10, 40, 80 mM for 72 hours (n=4). AML-MSCs were used as control (n=3). (E) Fluorescence intensity calculated from DiBAC fluorescence staining of h-MSCs (n=3) incubated with 10 nM Ouabain or 40 mM K^+ gluconate for 3 days followed by incubation with control medium (Washout, WO) for additional 3 days respect to untreated-AML-MSCs (n=2). (F) Dose-time curve of DiBAC fluorescence intensity in AML-MSCs treated with Lubiprostone at 1, 10, 50 µM for 72 hours (n=3). Healthy-MSCs were used as control (n=3). (G) Dose-time curve of DiBAC fluorescence intensity in AML-MSCs treated with IVM at 0,1 and 1 µM for 72 hours (n=3). Healthy-MSCs were used as control (n=3). (H) DiBAC fluorescence intensity of AML-MSCs (n=3) incubated with 10 µM Lubiprostone or 1 µM IVM for 3 days followed by incubation with control medium (WO) for additional 3 days. Healthy-MSCs were used as control (n=3).

All histograms show mean ± SEM; **p*-value <0.05, ***p*-value <0.01, ****p*-value <0.001, ****p*-value <0.001.

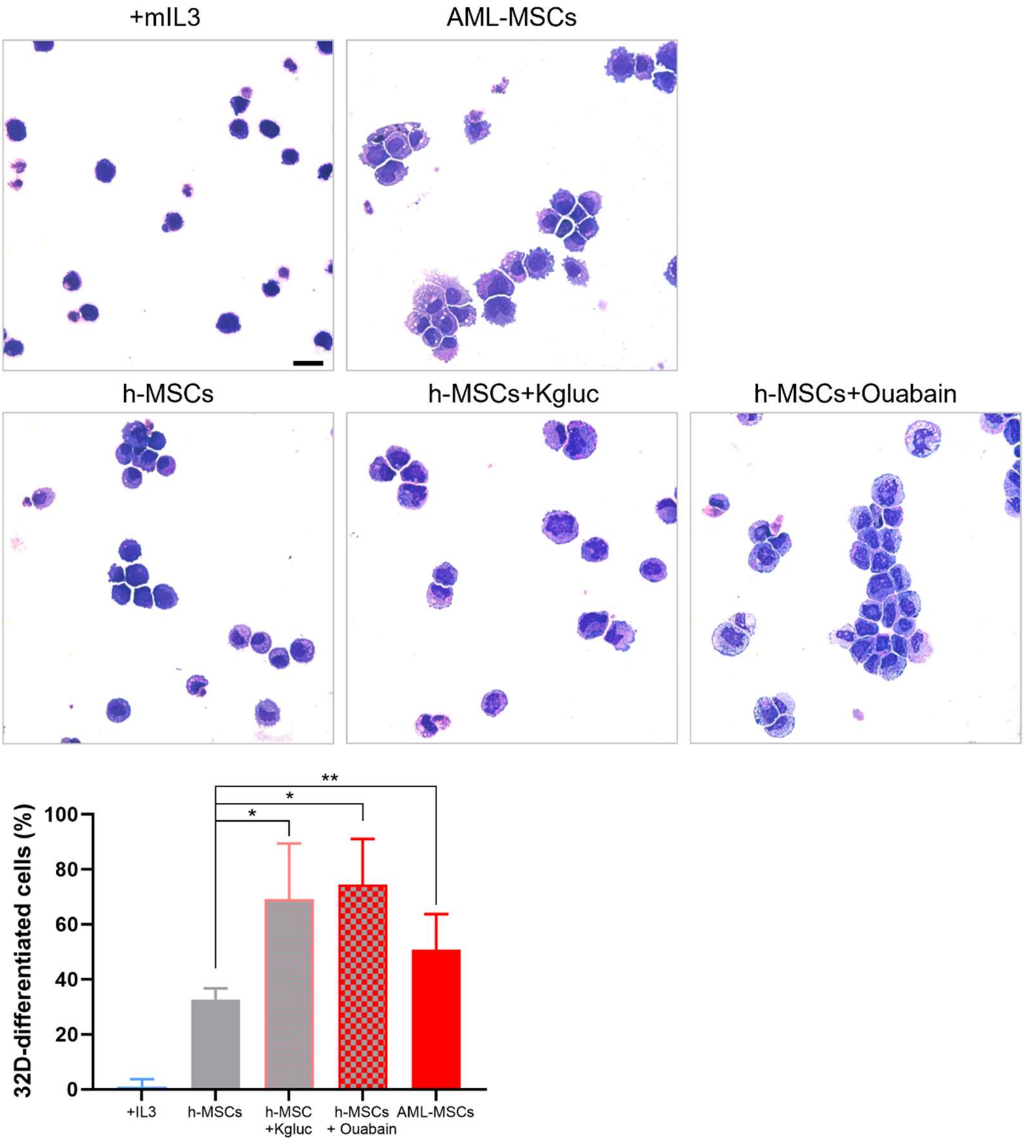

Supplementary Figure S2. MSCs role in 32D cell differentiation. Upper panel: Representative May-Grünwald Giemsa staining of 32D after a 72-hour co-culture with AML-MSCs, h-MSCs treated or not with K+ gluconate or Ouabain. Scale bar=10 µm. Lower panel: histogram reporting the percentage of differentiated 32D cells (n=5 pictures per condition, minimum 100 cells analyzed). Murine IL3 was used as experimental control.

The histogram shows mean ± SEM; **p*-value <0.05, ***p*-value <0.01.

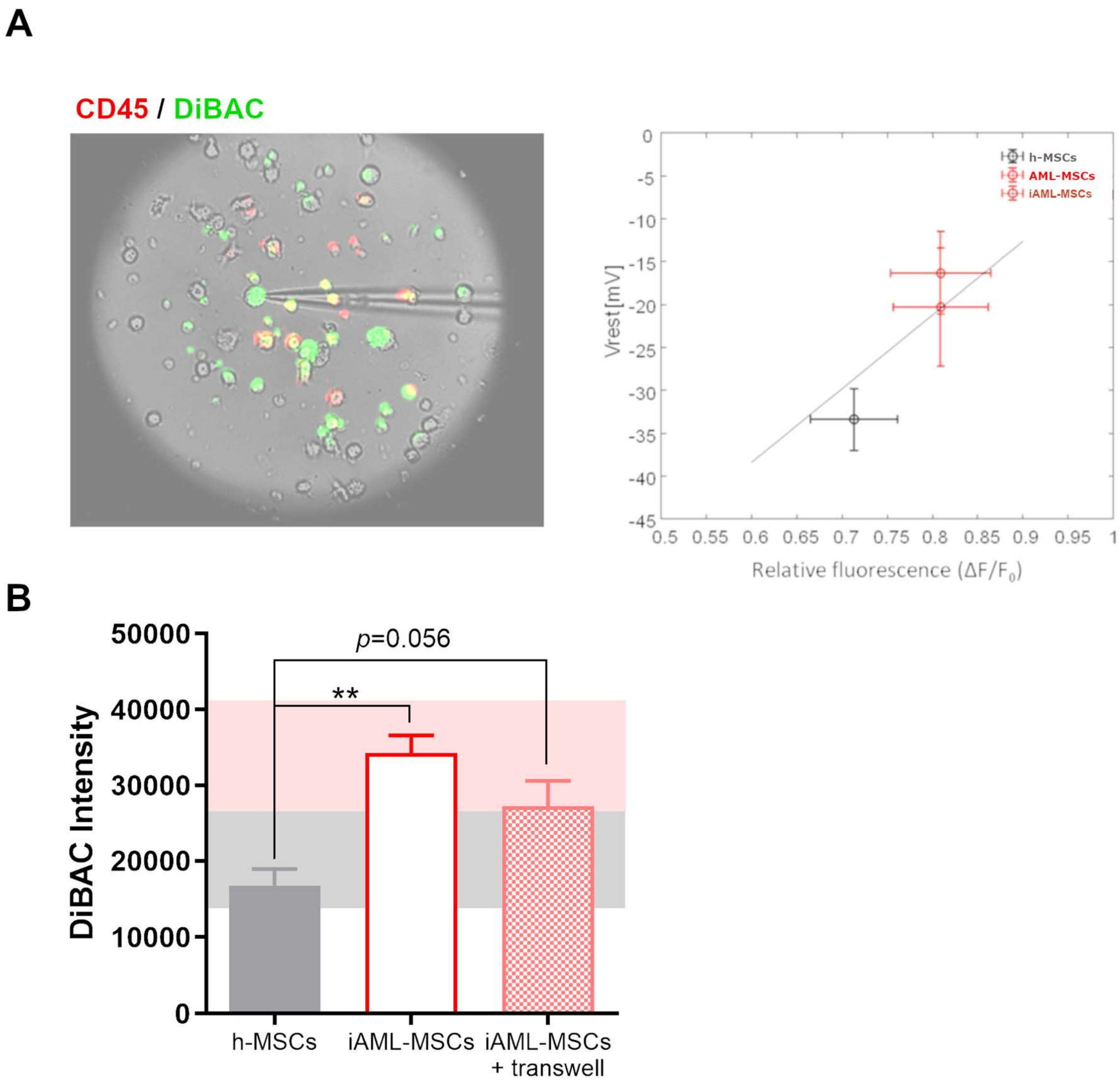

Supplementary Figure S3. AML blasts depolarize MSCs V_{mem}. (A) Representative image of patch clamp technique on co-culture dish with AML blasts ($CD45^+$, red) and MSCs loaded with DiBAC (green) (right panel) and linear regression model of mean fluorescence intensity $\Delta F/F_0$ of DiBAC and Vrest (mV) values obtained by patch clamp calculated for each group (left panel). (B) DiBAC V_{mem} measure of iAML-MSCs when a transwell insert was added during the co-culture with AML blasts (n=5, t-test comparing all groups *versus* h-MSCs). Healthy-MSCs were used as control.

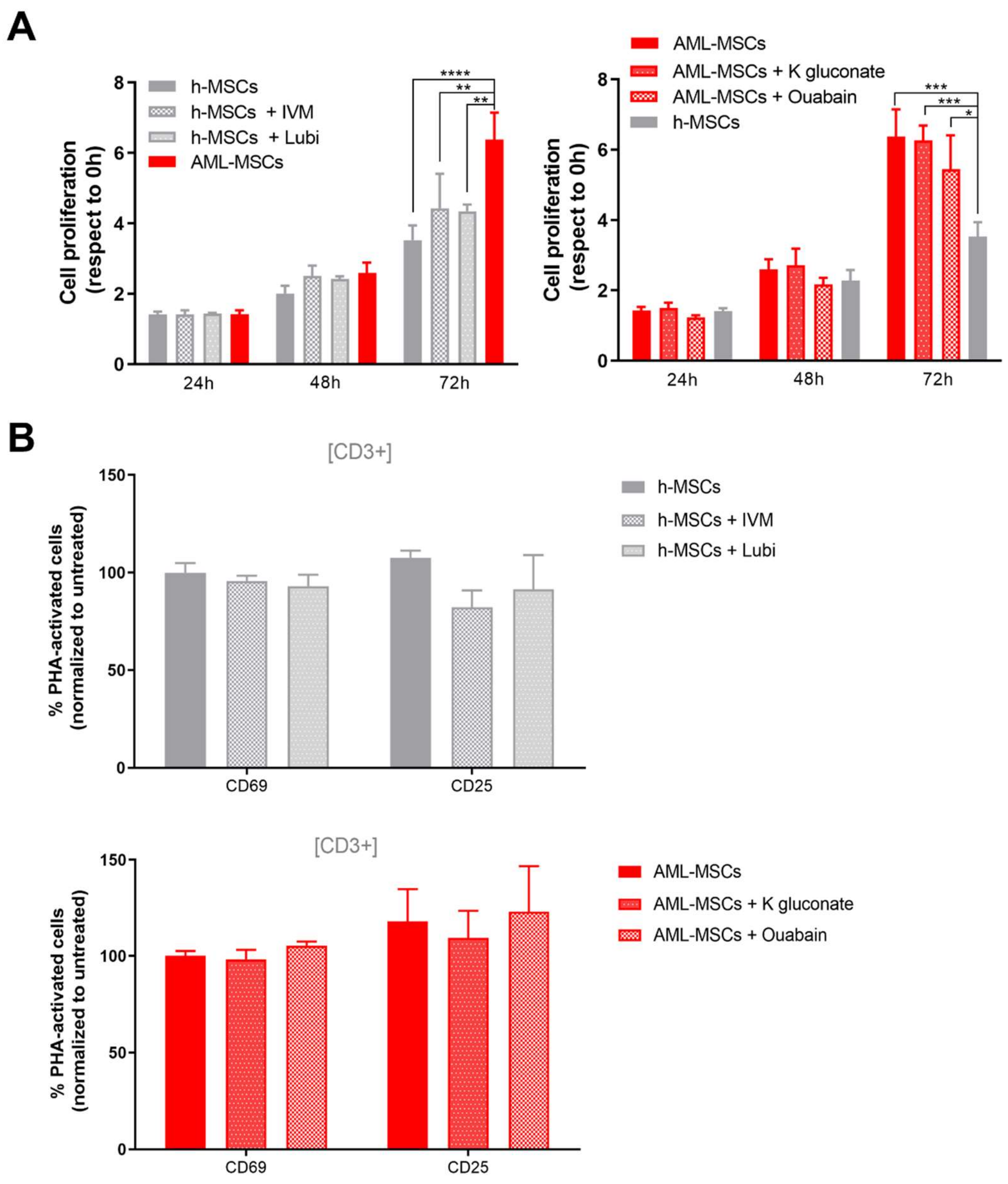

Supplementary Figure S4. Drug effects in MSCs. (A) Cell proliferation of h-MSCs, AML-MSCs, h-MSCs treated with 10 µM Lubi or 1 µM IVM and AML-MSCs treated with 40 mM K^+ gluconate or 10 nM Ouabain for 72 hours (by Presto Blue assay, n=3). Data were normalized to cell proliferation measured at 0 hours. (B) Percentage of PHA-stimulated $CD3^+$ T cells expressing CD69 and CD25 after 72 hours of co-culture with h-MSCs treated with Lubi (10 µM) or IVM (1 µM) (left panel) or AML-MSCs treated with 40 mM K^+ gluconate or 10 nM Ouabain (right panel), relative to untreated condition (n=3, p=not significant).

All histograms show mean ± SEM; *p-value <0.05, **p-value <0.01, ***p-value <0.001, ****p-value <0.0001.

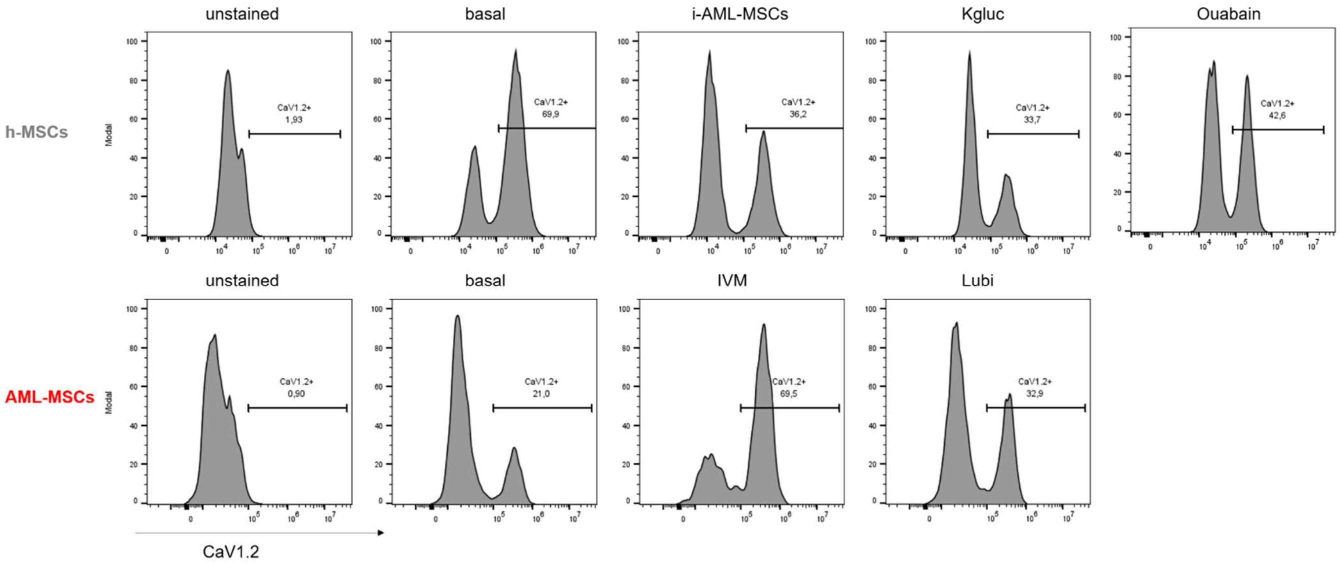

Supplementary Figure S5. V_{mem} changes modify CaV1.2 expression levels. Representative flow cytometry plots showing CaV1.2 expression on h-MSCs, h-MSCs co-cultured with AML blasts for 4 days (i-AML-MSCs) or h-MSCs treated with K^+ gluconate or Ouabain for 72 hours (upper panel). In the lower panel, histograms show CaV1.2 expression in AML-MSCs treated or not with Lubiprostone or IVM for 72 hours. Unstained samples are reported as control.

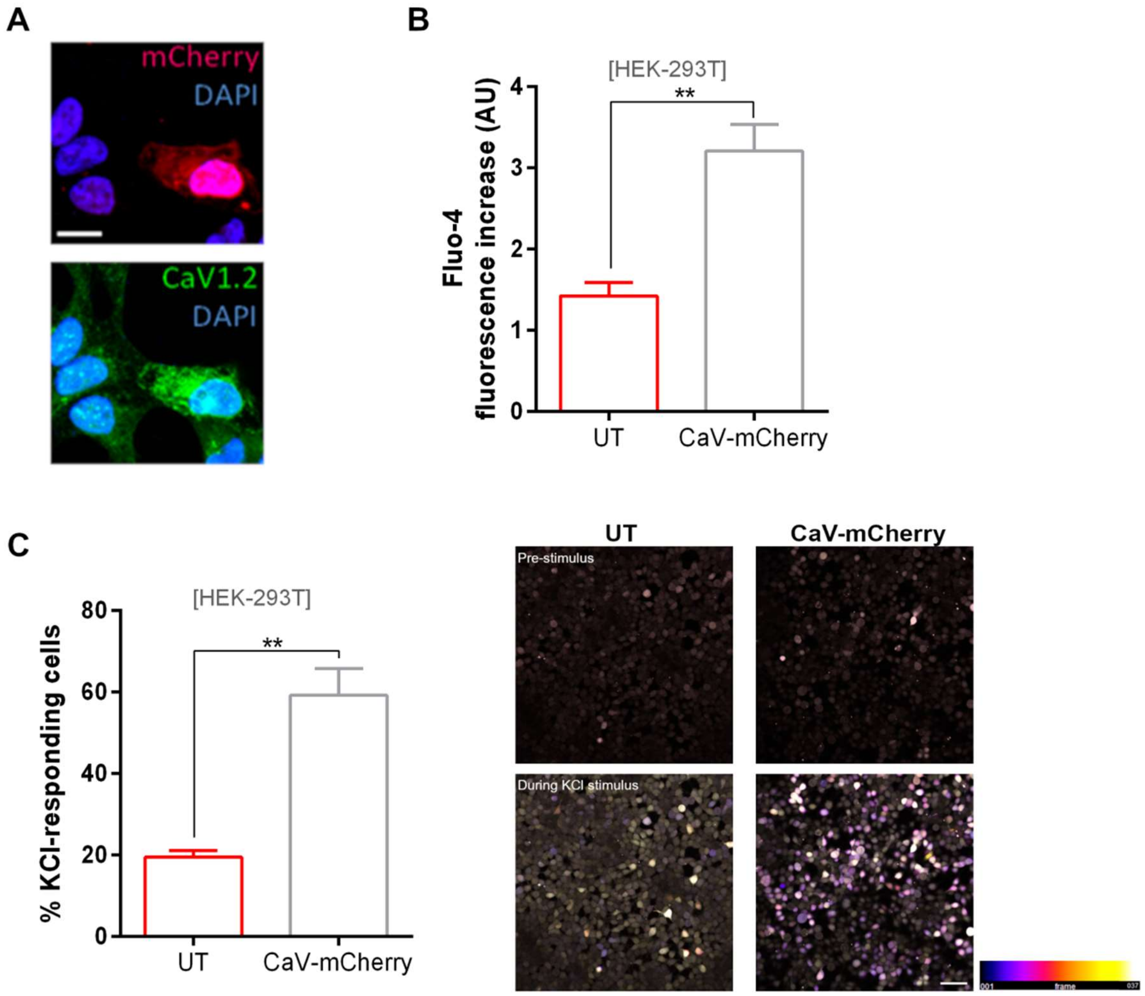

Supplementary Figure S6. Transient CaV1.2 overexpression controls calcium flux dynamics in HEK293T. (A) Immunofluorescence staining on HEK293T cells with CaV1.2 antibody at confocal microscopy after 48 hours of transfection with CaV1.2-mCherry (red) plasmid. CaV1.2 and DAPI staining is green and blue, respectively (scale bar=10 µm, n=1). (B) Intracellular calcium increases in un-transfected HEK293T (UT, n=27 cells) and CaV1.2-mCherry-expressing cells (n=37 cells), loaded with Fluo-4 AM probe and stimulated with KCl (65 mM). C) Percentage of KCl-responding cells (left panel) and representative time sequence (pre- and during KCl stimulus) of intracellular calcium increase (right panel) in UT and CaV1.2-mCherry-expressing HEK293T cells loaded with Fluo-4 AM dye (n=4).

All histograms show mean ± SEM; ***p*-value <0.01.

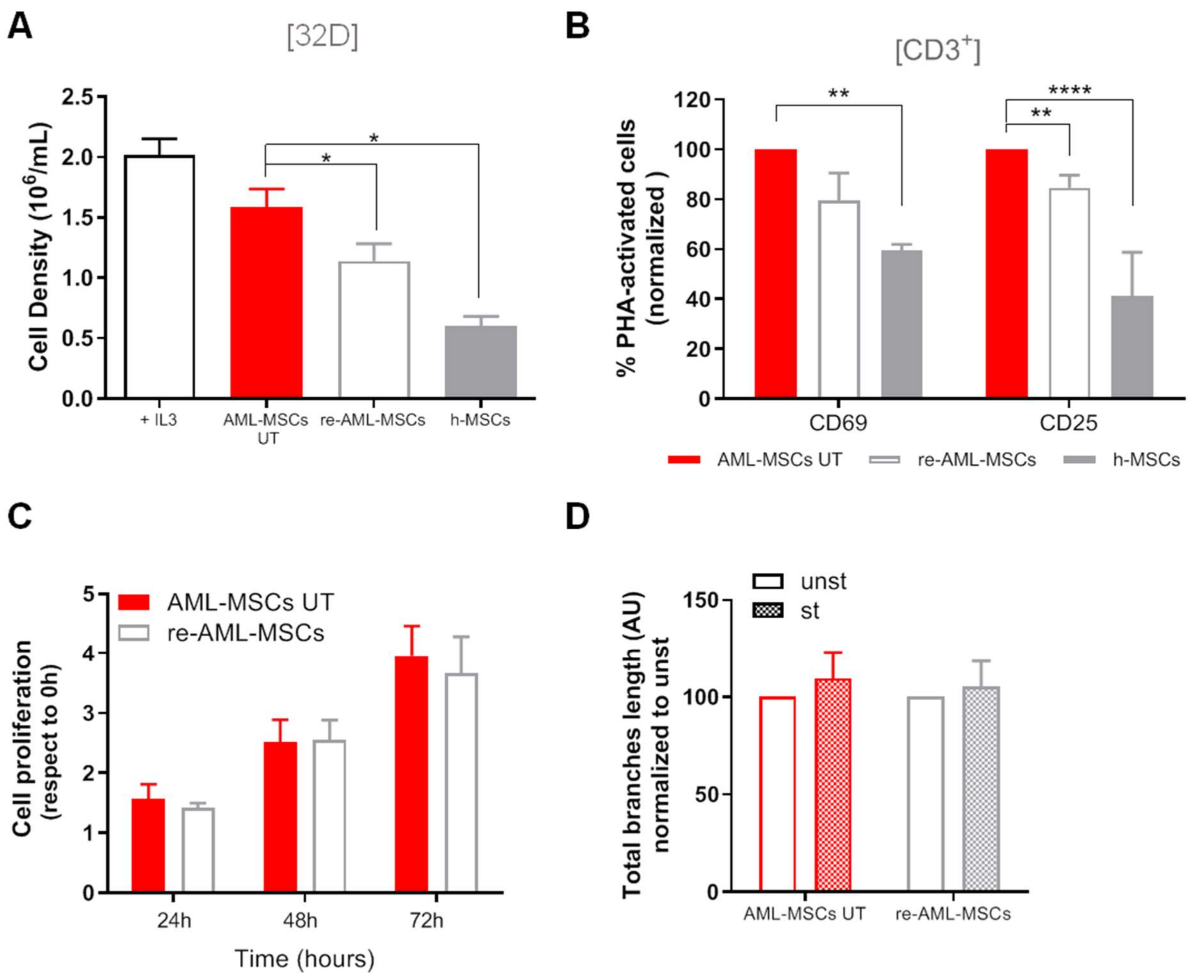

Supplementary Figure S7. CaV1.2 overexpression induces changes in MSCs phenotype. (A) Cell density of murine IL-3–dependent 32D cell line cultured for 72 hours with IL-3 or on a layer of AML-MSCs UT, re-AML-MSCs (n=9) or h-MSCs (n=2, t-test comparing all groups *versus* AML-MSCs; +IL3 used as experimental control). (B) Percentage of PHA-activated T-cells expressing CD69 and CD25 markers after 72 hours of co-cultured with AML-MSCs UT, re-AML-MSCs (n=6) and h-MSCs (n=3). Data were normalized to AML-MSCs UT condition. (C) Cell proliferation measured in AML-MSCs UT and re-AML-MSCs for 72 hours by Presto Blue assay (n=7). (D) Total length of branches formed by HUVEC endothelial cells when cultured with conditioned medium derived from AML-MSCs UT or re-AML-MSCs (n=7) stimulated (st) or not (unst) with a pro-inflammatory cytokines cocktail for 24 hours; tube formation was evaluated after 4 hours and normalized to unst condition (AU: arbitrary unit).

All histograms show mean ± SEM; *p-value <0.05, **p-value <0.01, ****p-value <0.0001.

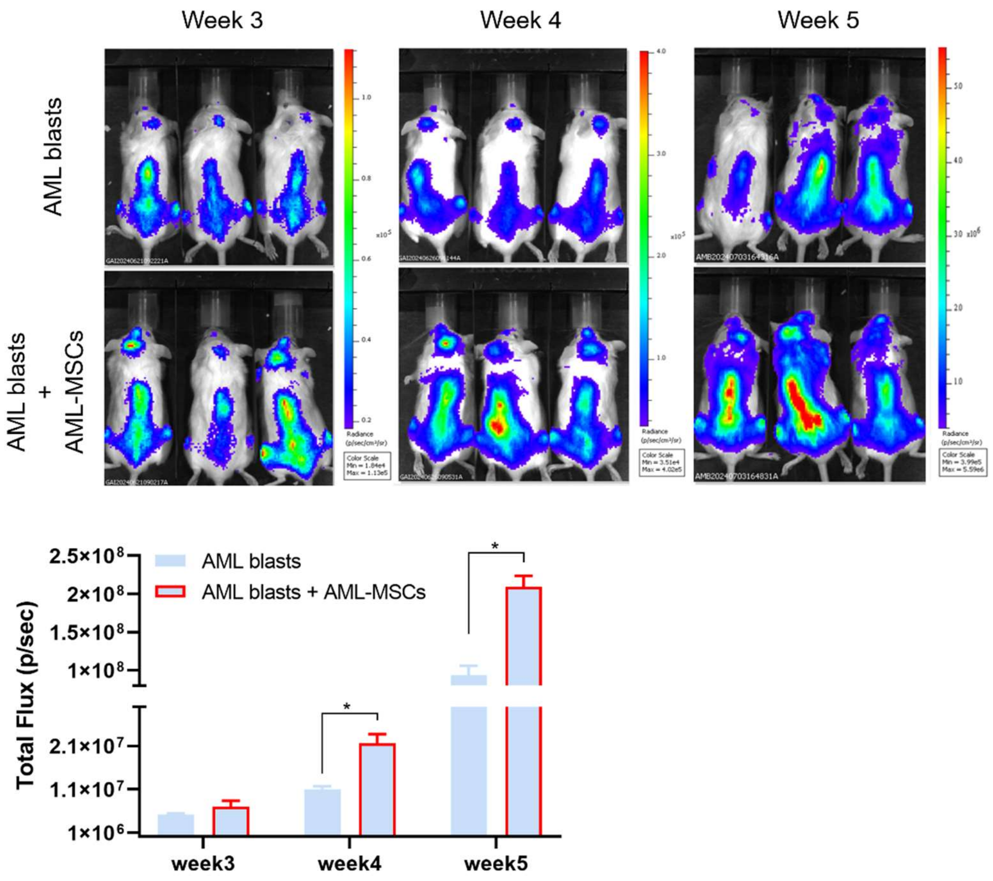

Supplementary Figure S8. Sustainment of AML-MSCs to blasts. Tumor growth of luciferase-transduced primary AML cells injected in NSG mice with or without AML-MSCs, measured by luminescence activity (left panel). Images of bioluminescence in mice (right panel, n=3 mice per group). All histograms show mean ± SEM; *p-value <0.05.

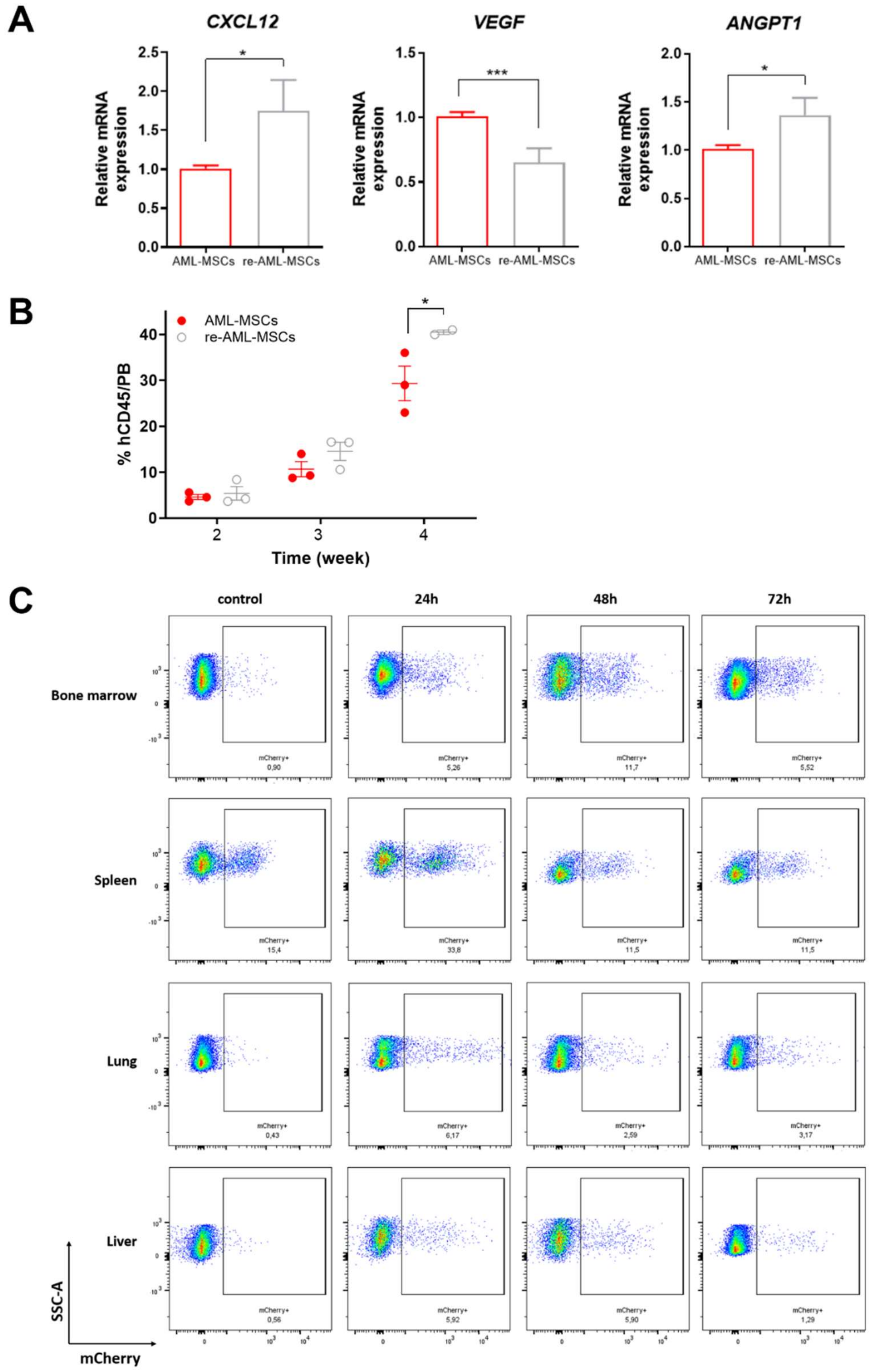

Supplementary Figure S9. MSCs influences normal hematopoiesis *in vivo*. (A) Expression analysis of BM niche-associated genes (*CXCL12*, C-X-C motif chemokines ligand 12, n=6; *VEGFA*, vascular endothelial growth factor A, n=7; *ANGPT1*, angiopoietin 1, n=8) in AML-MSCs and re-AML-MSCs. Results are expressed as ΔΔCT relative to AML-MSCs UT control condition. (B) Percentage of human CD45 cells in NOG-EXL mice PB at 2, 3, and 4 weeks after tail vein co-

infusion of $1.0x10^5$ human CB $CD34^+$ cells with $1.0x10^6$ AML-MSCs or re-AML-MSCs (n=3 mice per group). (C) Representative flow cytometry plots showing the tracking of mCherry-transduced MSCs in NSG mice in different districts (bone marrow, spleen, lung and liver) at 24-48-72 hours after injection.

All histograms show mean ± SEM; *p-value <0.05, ***p-value <0.001.

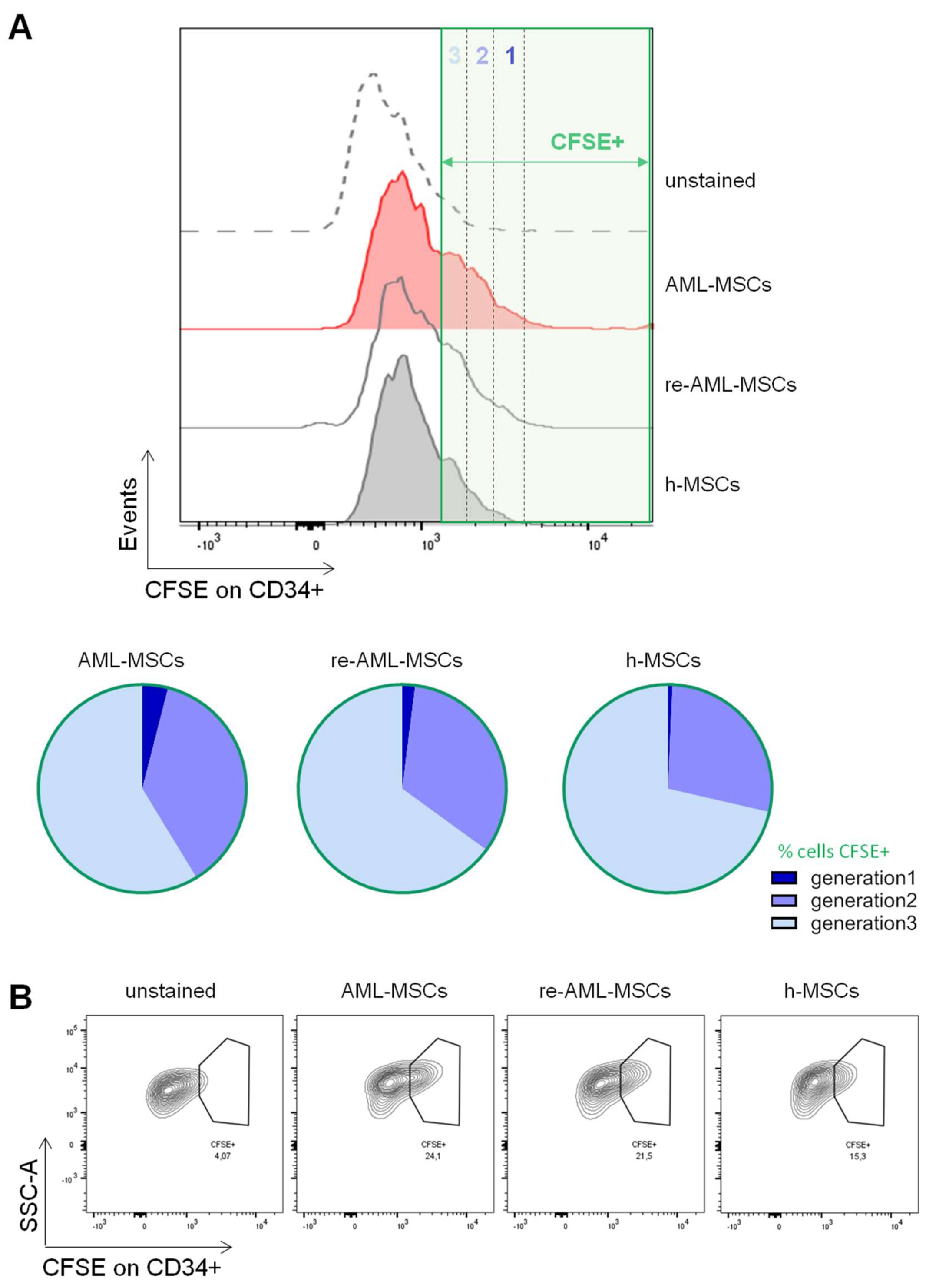

Supplementary Figure S10. Analysis of cell divisions in CD34+ cells by CFSE staining. (A) CFSE staining of CD34+ cells harvested from bone marrow of mice co-injected with AML-MSCs, re-AML-MSCs or h-MSCs, 72 hours after injection, highlighting different proportion of cells in the different cell generations. (B) Analysis of CFSE+ CD34+ cells at 72 hours post-injection in mice with AML-MSCs, re-AML-MSCs or h-MSCs.

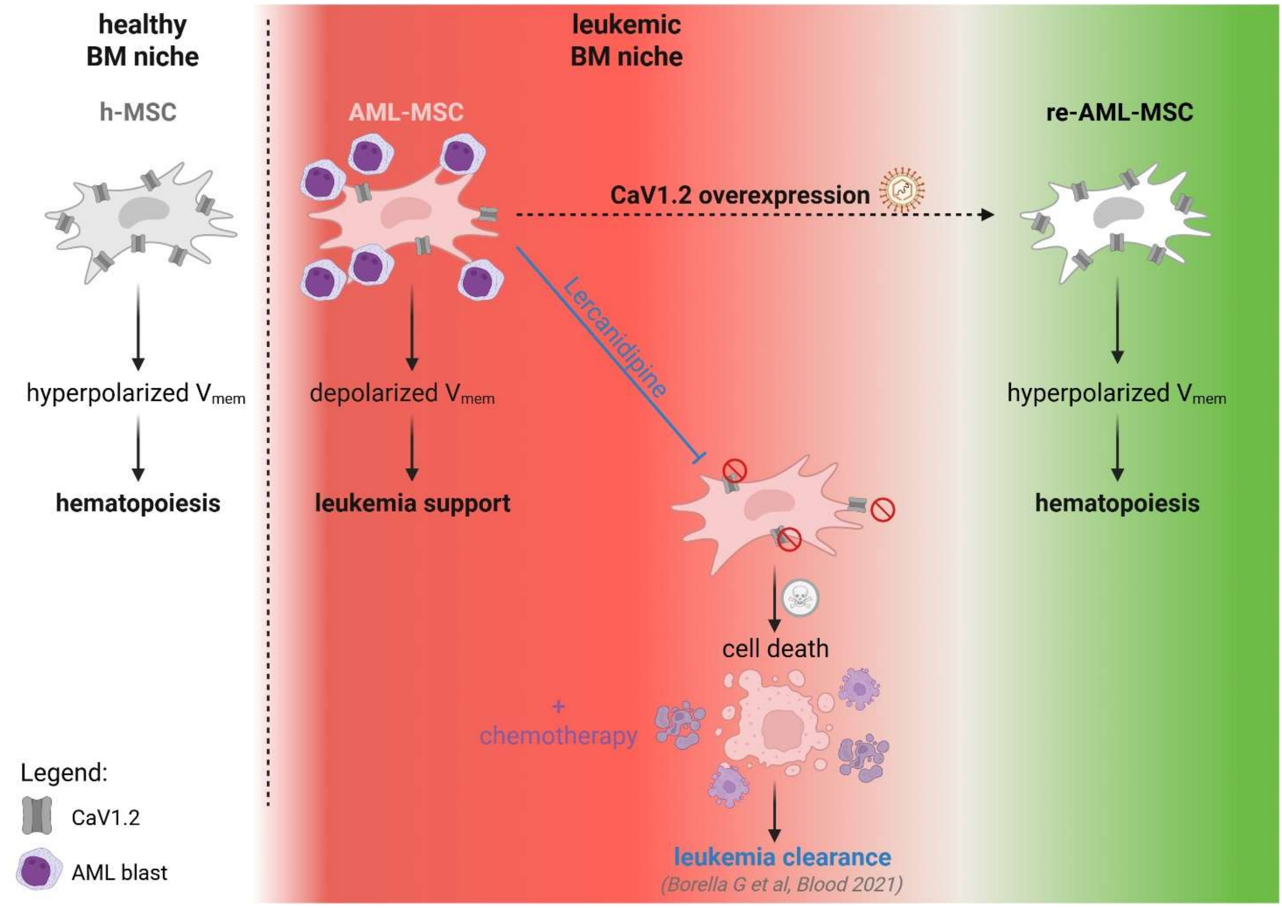

Supplementary Figure S11. Schematic model illustrating MSCs plasticity and therapeutic modulation in different context. In the healthy BM niche, h-MSCs exhibit a hyperpolarized V_{mem} that supports normal hematopoiesis. In contrast, AML-MSCs show lowered CaV1.2 expression, leading to depolarized V_{mem} and support of leukemic cells. Pharmacological inhibition of CaV1.2 with lercanidipine selectively induces AML-MSCs death, impairing leukemia-supportive stroma and enhancing chemotherapy-induced leukemia clearance. Restoration of CaV1.2 expression in AML-MSCs (re-AML-MSCs) re-establishes a hyperpolarized $V_{mem,}$ restoring a hematopoiesis-supportive microenvironment. Created with BioRender.com.
